# CXCR1 drives the pathogenesis of EAE and ARDS via boosting dendritic cells-dependent inflammation

**DOI:** 10.1038/s41419-023-06126-y

**Published:** 2023-09-14

**Authors:** Wei Zhuang, Jinfeng Zhou, Lan Zhong, Jie Lv, Xuan Zhong, Guangyu Liu, Ling Xie, Chun Wang, Kaidireya Saimaier, Sanxing Han, Changjie Shi, Qiuhong Hua, Ru Zhang, Xin Xie, Changsheng Du

**Affiliations:** 1 Key Laboratory of Spine and Spinal Cord Injury Repair and Regeneration of Ministry of Education, Orthopaedic Department of Tongji Hospital, School of Life Sciences and Technology, Tongji University, Shanghai, 200092 China; 2 Institute of Biophysics, Chinese Academy of Sciences, Beijing, 100101 China; 3 College of Life Sciences, University of Chinese Academy of Sciences, Beijing, 100049 China; 4 Department of Gastroenterology, Shanghai East Hospital, Tongji University School of Medicine, Shanghai, 200092 China

**Keywords:** Autoimmunity, Inflammasome

## Abstract

Chemokines secreted by dendritic cells (DCs) play a key role in the regulation of inflammation and autoimmunity through chemokine receptors. However, the role of chemokine receptor CXCR1 in inflammation-inducing experimental autoimmune encephalomyelitis (EAE) and acute respiratory distress syndrome (ARDS) remains largely enigmatic. Here we reported that compared with healthy controls, the level of *CXCR1* was aberrantly increased in multiple sclerosis (MS) patients. Knockout of CXCR1 not only ameliorated disease severity in EAE mice but also suppressed the secretion of inflammatory factors (IL-6/IL-12p70) production. We observed the same results in EAE mice with DCs-specific deletion of CXCR1 and antibody neutralization of the ligand CXCL5. Mechanically, we demonstrated a positive feedback loop composed of CXCL5/CXCR1/HIF-1α direct regulating of IL-6/IL-12p70 production in DCs. Meanwhile, we found CXCR1 deficiency in DCs limited IL-6/IL-12p70 production and lung injury in LPS-induced ARDS, a disease model caused by inflammation. Overall, our study reveals CXCR1 governs DCs-mediated inflammation and autoimmune disorders and its potential as a therapeutic target for related diseases.

## Introduction

Inflammation is one of the most important components of the immune system because of its critical role in physiological response to injury and infection. However, unresolved or aberrantly activated inflammation can become pathogenic [1]. The pathogenic inflammation triggers complex, self-perpetuating immune responses and activates of receptors on immune cells. Derived signalling from activated receptor cascades that amplify local or systemic immune responses compromise the structural integrity and/or function of specific cell types, tissues, or organs [2–4]. Previous evidence has revealed that some inflammatory disorders (acute respiratory distress syndrome (ARDS), inflammatory bowel disease (IBD), and autoimmune diseases (type 1 diabetes, primary biliary cirrhosis, and multiple sclerosis (MS)) are associated with a cytokine storm (characterized by high levels of IL-­6, IL­12p70, and IL-­1β, and tumor necrosis factor (TNF)) [5–7]. However, the massive production of inflammatory cytokines depends on the context, location, and timing, which makes it considerably difficult to develop effective therapeutics [8].

Dendritic cells (DCs) are the key link between innate immunity and adaptive immunity because of their promotion of immune defense and the maintenance of immune tolerance [9, 10]. On stimulation by antigens, DCs increase the expression of surface co-stimulatory molecules and pro-inflammatory or anti-inflammatory cytokines to direct differentiation of naïve CD4^+^ T cells [11–13]. Alterations in the functioning of DC may result in an imbalance of immune responses and even immune pathologies, like systemic lupus erythematosus (SLE), IBD, and MS. Thus, it is important to study molecular mechanisms that mediate DC activation and cytokines secretion function for identifying the underlying mechanisms [14–16]. Existing studies have reported that DCs produce polarizing and pro-inflammatory cytokines by complicated intracellular signaling pathways involving PI3K/AKT, MAPK/NF-κB, and JAK/STAT [17–20]. Furthermore, Beth et al. [21] suggested that transcription factor hypoxia-inducible factor-1α (HIF-1α) promotes DCs maturation and increases inflammatory gene expression as a metabolic switch in LPS-activated DCs. Although the intrinsic pathways of DCs secretion function have been well described, how instructive signals trigger and amplify the inflammatory responses in DCs remains poorly understood.

Chemokines are a family of inflammatory cytokines with the ability to regulate cell migration, adhesion, phagocytosis, cytokine secretion, proliferation, and apoptosis [22]. Increasing evidence suggests that chemokines and their receptors play a fundamental role in regulating DCs migration and homeostatic and inflammatory functions under both steady-state and inflammatory conditions [23–26]. However, very little is known about specific chemokines that mediate inflammatory cytokine by DCs in autoimmune diseases. Therefore, we focused on CXCR1, the first chemokine receptor cloned in 1991, and its mouse ligand CXCL5/LIX, which has been shown to mediate cell migration and granule release in neutrophils in response to bacterial infections and autoimmune diseases [27, 28]. Nevertheless, whether and how the CXCR1 plays critical roles in DCs-mediated inflammation and autoimmune disorders remains to be explored.

Herein, we found the mRNA level of *CXCR1* was markedly upregulated in the peripheral blood leukocytes of MS patients. The deletion of CXCR1 and inhibition of CXCL5 significantly reduced the production of inflammatory cytokines, including IL-6 and IL-12p70, by DCs and restricted EAE progression. Moreover, activation of CXCR1 signaling amplified inflammation production through the CXCL5/CXCR1/HIF-1α positive feedback in DCs. Significantly, CXCR1 was also found to be functionally involved in LPS-induced ARDS. Therefore, this study reveals the role and mechanism of CXCR1 in regulating DCs mediating inflammation and autoimmunity. Furthermore, our data also shed light on uncovering new strategies for the treatment of autoimmune and inflammatory diseases.

## Materials and methods

### Human subjects

The blood samples of MS patients and healthy donors were obtained from Shanghai East Hospital, affiliated with Tongji University in Shanghai. All procedures followed were in accordance with the ethical standards of the responsible committee on human experimentation (the institutional review board at Tongji University) and with the Helsinki Declaration of 1975, as revised in 2000. Informed consent was obtained from all subjects to be included in the study. All patient data were anonymized before study inclusion. See Table 1 for full patient information, including age, sex, and health status. All MS participants were examined for expanded disability status scale measures (EDSS) [29]. Informed consent was obtained from all subjects to be included in the study. All patient’s data were anonymized before study inclusion.

Peripheral blood mononuclear cells (PBMC) were isolated from the fresh peripheral blood of healthy volunteers (Stem Express, Inc.) by density gradient centrifugation using Ficoll Paque PLUS (GE Healthcare). CD14^+^ monocytes were purified by magnetic selection (Stem Cell Technology) according to the manufacturer’s protocol and 10^6^ cells/ml of CD14^+^ monocytes were cultured in complete RPMI 1640 supplemented with 10% FBS, l-glutamine (2 mM), 2-ME (50 mM), human recombinant GM-CSF (20 ng/ml), and human recombinant IL-4 (1 ng/ml) for 7 days. Thereafter, the cells were collected and seeded into 24-well plates (1 × 10^6^ cells per well per milliliter) in the presence of anti-CXCR1 (3, 10, and 30 μg/ml) and KC7F2 (HIF-1α inhibitor) [30] for 30 min.

### Mice

All animal experiments were approved by the Institutional Animal Care and Use Committee of Tongji University. OT-II C57BL/6J mice were obtained from Zhongjun Dong, Tsinghua University. GFP-OT-II C57BL/6J mice were generated by breeding GFP mice with OT-II mice. *Cxcr1*^−/−^ C57BL/6J mice were constitutive knockout mice and generated by TALNEN-mediated gene targeting, DC(WT) C57BL/6J mice were purchased from GemPharmatech Co., Ltd (T051927). Conditional deficiency of CXCR1 in DCs was generated by crossing *Cxcr1*^fl/fl^ mice with Itgax-cre C57BL/6J mice, which were purchased from The Jackson Laboratory (stock No. J008068). All mice were bred and maintained in the laboratory animal center of Tongji University in a specific pathogen-free (SPF) facility at room temperature and were 6–10 weeks at the beginning of the experiments.

### Cell line

HEK293T cell line was provided by P. Kang (School of Life Sciences and Technology, Tongji University, Shanghai, China). HEK293T cells were grown in Dulbecco’s modified Eagle’s medium (DMEM), supplemented with 5% (v/v) FBS and 1% (v/v) penicillin/streptomycin at 37 °C in 5% CO_2_ at 100% humidity.

### Reagents

MOG_35–55_ was purchased from GL Biochem with a purity > 95%. Complete Freund’s adjuvant (CFA) was purchased from Sigma-Aldrich. Anti-mouse CXCL5 antibody (MAB433–500) and isotype control (MAB0061) were purchased from R&D. Anti-mouse CD4-PE-Cy7 (1/100, 25-0042-82) was purchased from eBioscience. anti-mouse FOXP3-PE (1/100, 126404), anti-mouse IL-17A-PE (1/100, 506904), anti-mouse IFN-γ-APC (1/100, 505810) were purchased from BioLegend. OVA_323–339_ and lipopolysaccharides (LPS) from *Escherichia coli* O55:B5 (LPS 055: B5, L2880) were purchased from Sigma. Anti-mouse HIF-1α-PE (1/100, IC1935P) was purchased from R&D. U0126 (A1337) was purchased from Apexbio Technology. CoCl_2_ and KC7F2 were purchased from Sigma. Anti-mouse CXCR1 antibody (MAB330-100) was purchased from R&D.

### EAE model

Male mice at 10–12 weeks of age were immunized with MOG_35–55_ (200 μg) in CFA containing 5 mg/ml heat-killed mycobacterium tuberculosis H37RA. Mice received 200 ng of pertussis toxin (PTX, Calbiochem) by i.p. injection on days 0 and 2 [31]. Clinical appearance was assessed daily and scored as follows: 0, no clinical signs; 1, paralyzed tail; 2, paresis; 3, paraplegia; 4, paraplegia with forelimb weakness or paralysis; and 5, moribund or death. Mice received isotype control or anti-CXCL5 antibody by i.p. injection on days 0, 4, 8, 12, and 16.

After 3–4 weeks, all the mice were euthanized, mononuclear cells in the central nervous system (CNS) were removed for flow cytometry analysis, lumbar spinal cord was collected for hematoxylin and eosin (H&E) staining or luxol fast blue (LFB) staining and histology analysis.

### LPS-induced ARDS

Ten to twelve weeks-old male C57BL/6 mice, *Cxcr1*^−/−^, DC (WT), and DC (*Cxcr1*^−/−^), were subjected to LPS-induced ARDS as described [32, 33]. Briefly, 10 mg/kg LPS or vehicle was i.p. injected. Survival was monitored for up to 6 days, and survival rate was determined by Kaplan–Meier analysis. For acute lung injury, mice were injected with i.p. of 10 mg/kg LPS. Subsequently, 24 h after the LPS instillation, mice were euthanized, and bronchoalveolar lavage fluid (BALF) was collected with 1 ml of PBS and then centrifuged at 1000*g* for 10 min. Leukocytes in BALF were processed for CD4^+^IFN-γ^+^IL-17A^−^ (Th1 cells) and CD4^+^IFN-γ^−^IL-17A^+^ (Th17 cells) by flow cytometry. Multiple soluble cytokines: IL-6, CXCL5, and IL-12p70 in BALF supernatant, were measured by ELISA kits.

To obtain lung lymphocytes, lung tissue was minced into 1–2 mm pieces and enzymatically digested for 30 min at 37 °C in 15 ml of digestion buffer (RPMI 1640, 5% fetal calf serum, 100 U/ml penicillin, 100 μg/ml streptomycin sulfate, 1 mg/ml collagenase, 30 μg/ml DNase). After erythrocyte lysis using RBC lysis for 3 min, cells were washed, filtered over 70 μm cell strainer, and centrifuged for 30 min at 2000×*g* in the presence of 20% Percoll (Sigma-Aldrich) to separate leukocytes from cell debris and epithelial cells [34]. One leukocyte sample was isolated and processed for CD4^+^IFN-γ^+^IL-17A^−^ (Th1 cells) and CD4^+^IFN-γ^−^IL-17A^+^ (Th17 cells) by flow cytometry, and the other was isolated for the mRNA level of *Cxcl5*, *Hif1α*, *Il12a*, and *Il6* by qRT-PCR.

In all cases, at the experimental endpoint, the lungs were inflated with cold PBS, excised, and fixed in 4% paraformaldehyde (pH 7.2) for histological evaluation.

### Histopathology

Mice were euthanized and perfused with 25 ml of PBS with 2 mM EDTA by heart puncture to remove blood from internal organs. Cervical spinal cords or lungs were collected, fixed by 10% neutral buffered formalin solution, and decalcified. Tissues were then embedded in paraffin, sectioned, and stained with standard histological methods for hematoxylin and eosin (H&E) staining for inflammation. Cervical spinal cords stained with LFB staining for demyelination.

### CD4^+^ T cell isolation and differentiation in vitro

The methods were prepared as previously reported [35]. Briefly, splenocytes from 6–8week-old WT mice and *Cxcr1*^−/−^ mice were obtained and purified Naïve CD4^+^ T cells by a CD4^+^ T cells sorting kit (Dynabeads® Untouched^TM^ Mouse CD4 Cells Isolation Kit, Invitrogen). Cells were activated with anti-CD28 (2 μg/ml) and anti-CD3 (2 μg/ml) and then differentiated under Treg, Th1, or Th17 differentiation conditions. For Treg differentiation, hTGF-β1 (5 ng/ml), rmIL-2 (10 ng/ml), and anti-interferon gamma (anti-IFN-γ) (10 μg/ml) were added. For Th1 differentiation, anti-IL-4 (10 μg/ml) and IL-12 (10 ng/ml) were added. For Th17 differentiation, TGF-β1 (3 ng/ml), anti-IFN-γ (10 μg/ml), anti-IL-4 (10 μg/ml), IL-6 (30 ng/ml), IL-1β (10 ng/ml), and TNF-α (10 ng/ml) were added. Cells were collected on the fourth day for flow cytometry analysis.

### DCs and T cells co-culture

DCs were separated from the spleen of *Cxcr1*^−/−^ or WT mice with MOG_35–55_ induction on day 12 by using MagniSort® Mouse CD11c Positive Selection Kit (8802-6861-74, eBioscience). Naïve CD4^+^ T cells were purified from the spleen of *Cxcr1*^−/−^ or WT mice by using magnetic cell separation (Invitrogen). DCs and T cells were co-cultured at a ratio of 1:10. T cells were subjected to intracellular cytokine staining (ICS) and flow cytometry analysis on day 3 as follows: Th1 cells (CD4^+^ IFN-γ^+^) and Th17 cells (CD4^+^ IL-17A^+^). The supernatant was collected to measure the amount of IFN-γ, IL-17A, IL-6, IL-12p70, and TGF-β1.

To detect the proportion of T cell differentiation in co-culture after replenishing inflammatory factors (IL-6 and IL-12p70). For Th1 cell differentiation, WT and *Cxcr1*^−/−^splenic DCs were stimulated by LPS (100 ng/ml) for 12 hr, for washed and then cocultured with naïve CD4^+^ T cell in the presence of anti-IL4 (10 μg/ml) alone or supplemented with IL-12p70. After 3 days of co-culture, Th1 (CD4^+^ IFN-γ^+^) cells were analyzed by flow cytometry, and supernatants were collected to measure the amount of IFN-γ. For Th17 cell differentiation, WT and *Cxcr1*^−/−^ splenic DCs were stimulated by LPS (100 ng/ml) for 12 h, washed, and then cocultured with naïve CD4^+^ T cell in the presence of anti-IL4 (10 μg/ml) and anti-IFN-γ (10 μg/ml) or supplemented with IL-6. After 3 days of co-culture, Th17 (CD4^+^ IL-17A^+^) cells were analyzed by flow cytometry, and the supernatant was collected to measure the amount of IL-17A.

### In vitro permeability assay

For permeability assay, coculture transwell assay was performed using the 12-mm transwell with 0.4-mm pore size (Corning) In vitro. DCs were separated from the bone marrow of WT or *Cxcr1*^−/−^mice and cultured in complete RPMI 1640 supplemented with murine GM-CSF (20 ng/ml) and murine IL-4 (1 ng/ml) for 7 days. After 7 days, DCs (1 × 10^6^) were stimulated by LPS (100 ng/ml) for 24 h and precultured in the upper chamber of the Transwell. During the same time, naïve CD4^+^ T cells were purified from the spleen of WT mice by using magnetic cell separation (Invitrogen). In total, 3 × 10^5^ naïve CD4^+^ T cells were added to the bottom chamber and activated with anti-CD28 (2 μg/ml) and anti-CD3 (2 μg/ml), then differentiated under Th1 or Th17 differentiation conditions. For Th1 differentiation, anti-IL-4 (10 μg/ml) was added. For Th17 differentiation, anti-IFN-γ (10 μg/ml), and anti-IL-4 (10 μg/ml) were added. Cells were collected on the fourth day for flow cytometry analysis.

### Cell adoptive transfer

Naïve GFP OT-II T cells (2 × 10^6^) were injected into C57BL/6 mice by the lateral tail vein injecting for one day. In total, 2 × 10^5^ splenic DCs isolated from WT and *Cxcr1*^−/−^ mice were pulsed with 50 μg/ml OVA and 500 ng/ml LPS. After 8 h, the cells were then injected into recipient mice. Seven days later, mice were killed to analyze the Th1 cell (CD4^+^ IFN-γ^+^ IL-17A^−^) and Th17 cell (CD4^+^ IFN-γ^−^ IL-17A^+^) proportion in the spleen.

### Immunoblot analysis

Cells were lysed with SDS-PAGE loading buffer at 95 °C for 5 min and separated in 10% Bis–Tris gel, then transferred to PVDF membranes (Millipore) by electroblotting. The following primary antibodies were used: total-AKT (1/1000, CY5561), AKT phosphorylated at Ser473 (1/1000, CY6016) and Thr308 (1/1000, CY6017), ERK phosphorylated at T202/Y204 and T185/Y187 (1/1000, CY5277), total-JNK (1/1000, CY5490), GAPDH (1/1000, AB0037) were purchased by Abways Technology; total-ERK (1/1000, #9102), total-p38 (1/1000, #8690), p38MAPK phosphorylated at Thr180 and Tyr182 (1/1000, #4511), JNK phosphorylated at Thr183 and Tyr185 (1/1000, #4668), total-S6 (1/1000, #2217), S6 phosphorylated at Ser235/236 (1/1000, #4858) were purchased by Cell Signaling Technology, CXCR1 (1/500, 55450-1-AP) was purchased by Proteintech; anti-HIF-1α (1/1000, SC13515) was purchased by Santa Cruz Biotechnology.

### RNA isolation and qRT-PCR (quantitative real-time PCR)

For qRT-PCR were subjected as previously described [36]. Briefly, total RNA was isolated using TRIzol reagent (Molecular Research Center, Inc.) and subjected to cDNA synthesis using M-MLV Reverse Transcriptase (Promega) with random hexamer primers. Gene expression was analyzed by real-time PCR performed with Biotool^TM^ 2× SYBR Green qPCR Master Mix. The expression of individual genes was calculated with a standard curve method and normalized to the expression of GAPDH. The gene-specific PCR primers (all mouse and human genes) are shown in Supplementary Table 1.

### Enzyme-linked immunosorbent assay

Cytokines in culture supernatants after in vitro stimulation, in BALF, and in peripheral blood were quantified by ELISA according to the manufacturer’s instructions as follows: for mouse IL-17A, IL-6, IL-12p70, TGF-β1, and IFN-γ, Invitrogen; for mouse CXCL5/LIX, R&D Systems; for human IL-6, IL-12p70, Invitrogen.

### Chromatin immunoprecipitation

The chromatin immunoprecipitation (ChIP) assay was performed with lysates from bone marrow-derived DCs after LPS stimulation by using SimpleChIP® Enzymatic Chromatin IP Kit (Cell Signaling Technology). Anti-HIF-1α (Cat. #14179, Cell Signaling Technology) was used for the ChIP assay.

The following primer pairs were used:

*Cxcl5* HRE-1 Forward: 5′-TGCCAAAACTAATTTGTATGCTGT-3′

Reverse: 5′-CTGGGGAGTGGTAATCTGTAACAA-3′

*Cxcl5* HRE-2 Forward: 5′-TCATCTGCCCCAAGGAAGCCAAAA-3′

Reverse: 5′-ATGCAGGACCACATTGCCACATTG-3′

*Vegf* Forward: 5′-CTGGGCAGCTGGCCTACCTACCTT-3′

Reverse: 5′-GTTAGTCAGTGGCGGGGAGTGAGA-3′.

### Plasmids

The mCXCL5 promoter was amplified from genomic DNA isolated from splenocyte of C57BL/6 mice using primers 5′-GGGGTACCATGACAAATGGATACAGGGAACAAC and 5′-GGAATTCCATATGTCCCGGACCAGGGAGCACCA and then subcloned into the pGL3 plasmid with KpnI and NdeI to create pGL3 mCXCL5 promoter-luciferase. The coding sequence of HIF-1α was amplified from the cDNA of mice splenocytes using primers 5′-CGGGATCCATGGAGGGCGCCGGCGGCGAGA and 5′-CGGAATTCCGCTCAGTTAACTTGATCCAAAGCT, then subcloned into the pcDNA3.0 plasmid with BamHI and EcoRI. The coding sequence of HIF-1β was amplified from the cDNA of mice splenocytes using primers 5′-CGGGATCCATGGCGGCGACTACAGCTAACCC and 5′-GCTCTAGATTCTATTCGGAAAAGGGGGGAA, then subcloned into the pcDNA3.0 plasmid with BamHI and XbaI.

### Transfection and luciferase assays

HEK293T cells were transfected with 2 μg of each plasmid by using the calcium phosphate precipitation method. A firefly luciferase reporter gene construct (200 ng per well) and pRL-SV40 Renilla luciferase construct (1 ng per well; for normalization) were co-transfected. Cell extracts were prepared 24 h after transfection, and luciferase activity was measured with the Dual-Luciferase Reporter Assay system (Promega). Wild-type or mutated 3′ UTR sequences of Ets1 or Smo (encoding smoothened) were cloned into the modified pGL3-control vector (Promega) as described [37].

### Statistical analysis

Statistical analysis was performed with GraphPad Prism (GraphPad Software). An unpaired, one-tailed parametric *t*-test was used for comparison of datasets. Data are presented as mean ± SEM. A Mann–Whitney *U* test was used to assess the significance of EAE clinical scores throughout the disease course. Spearman’s correlation coefficient, *R*^2^, was used to evaluate the association between the mRNA level of *Hif1α*, *Cxcr1*, and EDSS. A *p*-value of <0.05 was considered statistically significant.

## Results

### CXCR1 deficiency ameliorates EAE development

**Fig. 1:**
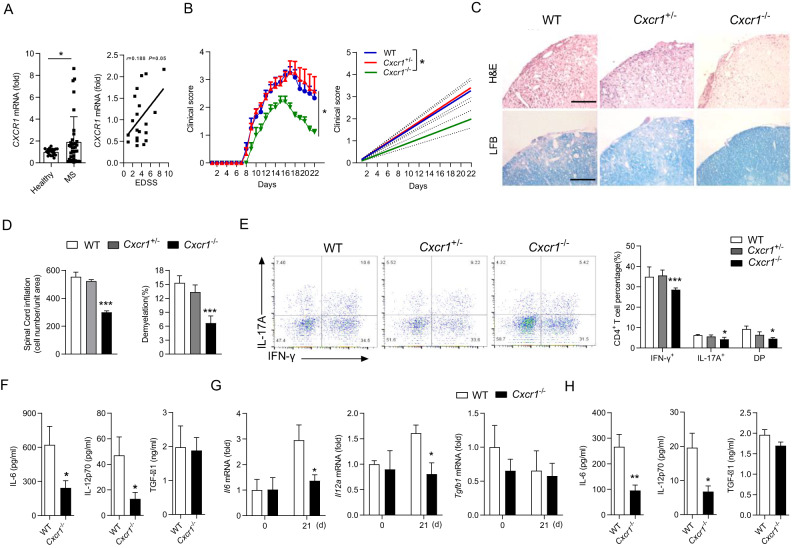
CXCR1 deficiency suppresses EAE development. **A** *CXCR1* mRNA expression in peripheral blood leukocytes in healthy controls (*n* = 21) and MS patients (*n* = 35) (left panel). Scatterplots showing the correlation between mRNA level of *CXCR1* and EDSS in MS patients (right panel). WT and *Cxcr1*^−/−^ mice were immunized with MOG_35–55_ peptides in CFA adjuvant and pertussis toxin to induce EAE. **B** Clinical scores of EAE in immunized WT and *Cxcr1*^−/−^ mice (left panel), and linear regression analysis (right panel) of the recipient mice depicted (*n* = 6). The data are expressed as the mean ± SEM. ^*^p < 0.05, vs. WT group (Mann–Whitney *U* test). **C** H&E staining and LFB staining of spinal cord paraffin sections from the WT and *Cxcr1*^−/−^ mice 28 days after EAE induction. Scale bars, 200 μm. **D** Pooled data are presented from (**C**). **E** Representative flow cytometry data showing intracellular production of IFN-γ and IL-17A in CD4^+^ T cells from the spinal cord and brain of WT and *Cxcr1*^−/−^ mice on 28 days after EAE induction. Pooled data are presented in the right panel. **F** ELISA analysis of cytokines (IL-12p70, IL-6, and TGF-β1) in the serum from WT and *Cxcr1*^−/−^ mice 12 days after EAE induction. **G** mRNA levels of *Il12a*, *Il6*, and *Tgfb1* in the brain from WT and *Cxcr1*^−/−^ mice on days 0 and 21 after EAE induction. **H** ELISA analysis of IL-6, IL-12p70, and TGF-β1 in supernatant of DCs sorted from WT and *Cxcr1*^−/−^ mice 12 days after EAE induction and restimulated with MOG_35–55_ for 48 h. Data are mean ± SEM. ^*^p < 0.05, ^**^p < 0.01, ^***^p < 0.001 vs. WT group (one-tailed Student’s *t*-test). Data are representative of three independent experiments with similar results.

To identify the role of CXCR1 in MS, we first analyzed the mRNA expression of *CXCR1* in peripheral blood leukocytes from 35 MS patients and 21 healthy donors (Table 1). The results indicated that *CXCR1* was significantly upregulated in MS patients and positively correlated with the EDSS (Fig. 1A). The data suggested potential involvements of CXCR1 in the pathogenesis of MS. We analyzed and found that mRNA expression and protein level of CXCR1 in CNS was significantly increased in the later period of the EAE progression (Supplementary information, Fig. S1A, B). Given the increased mRNA of *CXCR1* in MS patients and EAE mice, we aimed to gain insight into the role of CXCR1 in EAE development. Thus, we generated CXCR1-deficient mice (Supplementary information, Fig. S2), and CXCR1-deficiency showed no significant effects on the development of macrophages, DCs, B cells, CD4^+^ T cells, and CD8^+^ T cells (Supplementary information, Fig. S3). MOG_35–55_ was used to induce EAE, and results indicated that clinical score was significantly reduced in *Cxcr1*^−/−^ mice (Fig. 1B). Pathologically, CXCR1 deficiency significantly ameliorated the leukocyte infiltration, inflammatory foci, and demyelination (Fig. 1C, D). In addition, CD4^+^ T cells isolated from CNS of *Cxcr1*^−/−^ mice showed decreased expression of intracellular IFN-γ and IL-17A compared to those of WT mice (Fig. 1E). CD4^+^ T helper (Th) cells partly regulate immune response depending on the inflammation cytokine milieu [38, 39]. Therefore, we detected the inflammatory cytokines’ production in the serum of EAE mice, CXCR1 deficiency induced downregulation of IL-12p70 and IL-6, but not TGF-β1(Fig. 1F). Meanwhile, downregulated mRNA levels of *Il12a* and *Il6* was detected in CNS of *Cxcr1*^−/−^ mice compared with WT mice (Fig. 1G). It is well-known that DC plays a crucial role in CD4^+^ T cell differentiation via regulating the secretion of polarizing cytokines, such as IL-6, IL-12p70, and TGF-β1 [40]. We found that the expressions of IL-6 and IL-12p70, rather than TGF-β1, were significantly downregulated in splenic DC from *Cxcr1*^−/−^ mice (Fig. 1H). Therefore, these data confirmed that CXCR1 was important for promoting EAE progression.

**Table 1:** Characteristics of MS patients and healthy.

Group	Sample size	Age (year, mean ± SD)	EDSS	Clinical stage
Healthy controls	21 (15 F, 6 M)	43.4 ± 9.88	–	–
RRMS patients	35 (21 F, 14 M)	40.56 ± 12.19	4.7 ± 2.6	Relapsing

### CXCR1 augments the production of inflammatory cytokines in DCs

**Fig. 2:**
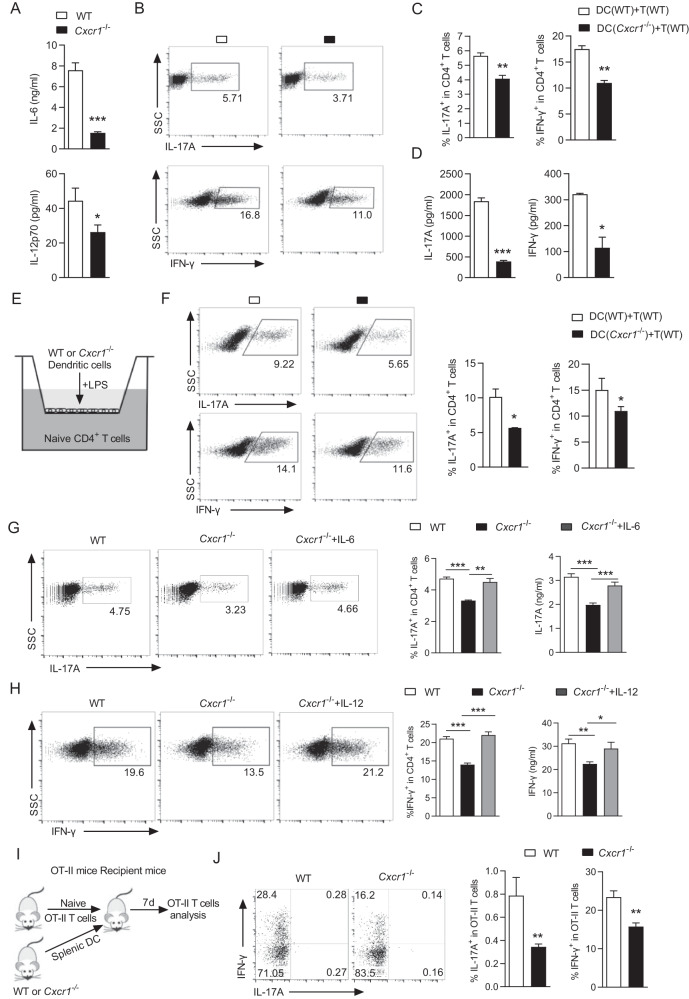
CXCR1 modulates the production of inflammatory cytokines in DCs. **A** DCs sorted from WT and *Cxcr1*^−/−^ mice and treated with LPS (100 ng/ml) for 24 h. IL-6 and IL-12p70 in DCs supernatant were examined by ELISA. **B** Representative flow cytometry data showing intracellular production of IFN-γ and IL-17A in CD4^+^ T cells from DC-T cell coculture system in vitro. **C** Pooled data are presented from (**B**). **D** ELISA detection of IFN-γ and IL-17A in supernatants of cocultured DC-T cells. **E** DCs from WT or *Cxcr1*^−/−^ mice were stimulated with LPS for 24 h and cultured in a transwell to naïve CD4^+^ T cell differentiation. **F** Representative flow cytometry data showing intracellular production of IFN-γ and IL-17A in CD4^+^ T cells. **G**, **H** The percentage of positive cells and protein levels of IL-17A (**G**) and IFN-γ (**H**) in cocultured DC-T cells or their supplemented. Pooled data are presented in the right panel. **I** Schematic diagram of cell adoptive transfer. **J** Representative flow cytometry data showing intracellular production of IFN-γ and IL-17A in CD4^+^ T cells 7 days after transfer. Pooled data are presented in the right panel. Data are mean ± SEM (*n* = 5–6). ^*^p < 0.05, ^**^p < 0.01, ^***^p < 0.001 vs. WT or indicated group. (one-tailed Student’s *t*-test). Data are representative of three independent experiments with similar results.

The indispensable role of CD4^+^ T cells in the pathogenesis of EAE prompted us to investigate whether CD4^+^ T cell differentiation was affected by CXCR1. To analyze whether CXCR1 has a direct effect on T cell differentiation, CD4^+^ T cell differentiation was induced under different polarizing conditions in vitro. Flow cytometric analysis revealed that CXCR1 did not affect Treg, Th1, and Th17 cell differentiation, suggesting that CXCR1 had no obvious effect on T cell differentiation directly in vitro (Supplementary information, Fig. S4). To unequivocally demonstrate the participation of the CXCR1 in the production of inflammatory cytokines in DCs, we first sorted the DCs from the spleen of wild-type mice and *Cxcr1*^−/−^ mice, then stimulated DCs with LPS. DCs from the spleen of *Cxcr1*^−/−^ mice showed lower expressions of IL-6 and IL-12p70 compared with that from WT mice (Fig. 2A). Further, we established a co-culture model of DCs and naïve CD4^+^ T cells to investigate the ability of CXCR1 to regulate DCs in activating and polarizing CD4^+^ T cells into specific subsets. DCs from *Cxcr1*^−/−^ and WT mice were activated with LPS and co-cultured with naïve CD4^+^ T cells. Analysis of intracellular IFN-γ and IL-17A expression revealed that *Cxcr1*-deficiency inhibited the ability of DCs to polarize CD4^+^ T cells differentiating into Th1 and Th17 (Fig. 2B, C) and found downregulated expressions of IFN-γ, IL-17A, IL-6, and IL-12p70 (Fig. 2D) (Supplementary information, Fig. S5). To gain insight into the direct roles of secreted cytokines (IL-6 and IL-12p70), we performed transwell experiment to test the T cell differentiation in presence of DCs with WT or *Cxcr1*-deficiency after LPS stimulated (Fig. 2E). The proportion of Th1 and Th17 as differentiation by the secreted cytokines from DCs into the bottom chamber significantly decreased by DCs with *Cxcr1*-deficiency (Fig. 2F).

To determine whether the impaired IL-6 and IL-12p70 production in *Cxcr1*^−/−^ DCs was related to the regulation of T cell differentiation, we applied the co-culture model of DCs and naïve CD4^+^ T cells and showed that additional supplementation of IL-6 and IL-12p70 significantly reversed CXCR1 deficiency-induced Th17 and Th1 differentiation impairment, respectively (Fig. 2G, H). To further evaluate the effects of CXCR1 in DCs required on CD4^+^ T cell differentiation in vivo, splenic DCs obtained from WT and *Cxcr1*^−/−^ mice were primed with LPS and OVA and then injected into two groups of recipient mice that previously received naïve GFP OT-II T cells (Fig. 2I). A significant decrease of Th17 and Th1 differentiation was observed in mice receiving DCs with CXCR1 deficiency (Fig. 2J). Together, these data emphasized that CXCR1 is a prerequisite to produce proinflammatory cytokines by DCs and mediate T cell differentiation.

### CXCR1 expression in DCs accelerates EAE progression

To further study the role of CXCR1 in DCs during EAE, we first assessed the expression of CXCR1 in DCs from WT mice with or without EAE. Early in the pathogenesis of EAE, DCs in the spleen were isolated from mice, and the mRNA level of *Cxcr1* by quantitative real-time PCR. Compared with the WT mice without EAE, we observed the upregulated level of *Cxcr1* mRNA level in WT mice with EAE (Supplementary information, Fig. S6). Thus, to determine whether CXCR1 in DCs has an important role in the regulation of EAE pathogenesis, we crossbred Itgax^cre^ transgenic mice to mice with CXCR1 loci flanked by loxp sequences (CXCR1^fl/fl^) to generate CXCR1^fl/fl^ Itgax^cre^ (termed DC (*Cxcr1*^−/−^) mice), in which CXCR1 was conditional knockout in DCs (Supplementary information, Fig. S7A, B). The percentage and absolute number of DCs, macrophages, B cells, CD4^+^ T cells, and CD8^+^ T cells were not altered upon CXCR1 deficiency (Supplementary information, Fig. S7C, D). Importantly, the mRNA level of *Cxcr1* in the brain of DC (*Cxcr1*^−/−^) mice with EAE was significantly lesser than that observed in WT mice with EAE but similar to that seen in the WT mice (Supplementary information, Fig. S8).

**Fig. 3:**
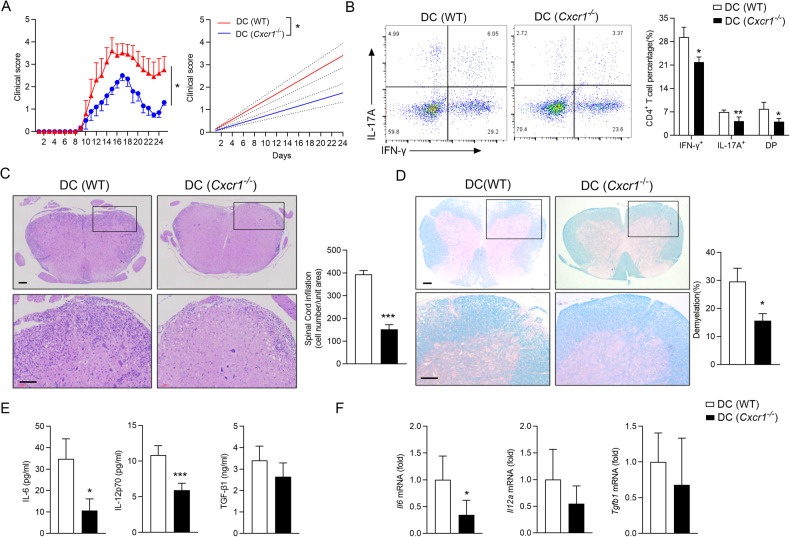
CXCR1 deficiency in DC ameliorates EAE progression. WT or DC conditional knockout mice (DC (WT) or DC (*Cxcr1*^−/−^)) were immunized with MOG_35–55_ peptides in CFA adjuvant and pertussis toxin to induce EAE. **A** Mean clinical scores of EAE in DC (WT) and DC (*Cxcr1*^−/−^) mice (left panel), and linear regression analysis (right panel) of the recipient mice depicted (*n* = 5). The data are expressed as the mean ± SEM. ^*^p < 0.05, vs. DC (WT) group (Mann–Whitney *U* test). **B** Representative flow cytometry data showing intracellular production of IFN-γ and IL-17A in CD4^+^ T cells from the spinal cord and brain of DC (WT) and DC (*Cxcr1*^−/−^) mice 28 days after EAE induction. Pooled data are presented in the right panel. **C** H&E staining and LFB staining (**D**) of spinal cord paraffin sections from the DC (WT) and DC (*Cxcr1*^−/−^) mice 28 days after EAE induction. Scale bars, 200 μm. Pooled data are presented in the right panel. **E** IL-12p70, IL-6, and TGF-β1 levels in the serum of DC (WT) and DC (*Cxcr1*^−/−^) mice 12 days after EAE induction were detected by ELISA. **F** Expression of *Il12a, Il6*, and *Tgfb1* mRNA in the spinal cord and brain from the DC (WT) and DC (*Cxcr1*^−/−^) mice 28 days after EAE induction. Data are mean ± SEM. ^*^p < 0.05, ^**^p < 0.01, ^***^p < 0.001 vs. DC(WT) group (one-tailed Student’s *t*-test).

As expected, DC (*Cxcr1*^−/−^) mice showed lower disease scores than DC (WT) group after immunization MOG_35–55_ (Fig. 3A). The expression of IFN-γ^+^ CD4^+^ T and IL-17A^+^ CD4^+^ T cells in CNS of DC (*Cxcr1*^−/−^) mice was lower compared to that in DC (WT) mice (Fig. 3B). Associated pathologies (leukocyte infiltration, inflammatory foci, and demyelination) were dramatically ameliorated in DC (*Cxcr1*^−/−^) mice (Fig. 3C, D). We further isolated serum from DC (*Cxcr1*^−/−^) and DC (WT) mice for 12 days in the EAE model, DCs specific CXCR1-deficiency downregulated the expression of IL-12p70 and IL-6, concomitant with no difference expression of TGF-β1 (Fig. 3E). QPCR analysis revealed that *Il6* mRNA level was downregulated significantly in spinal cord of mice with CXCR1-deficiency in DCs, whereas the expression of *Il12a* and *Tgfb1* remained unchanged (Fig. 3F). These results suggested that CXCR1 deficiency in DCs restricted the progression of EAE.

### CXCL5/CXCR1 axis is required for aggravating the severity of EAE

**Fig. 4:**
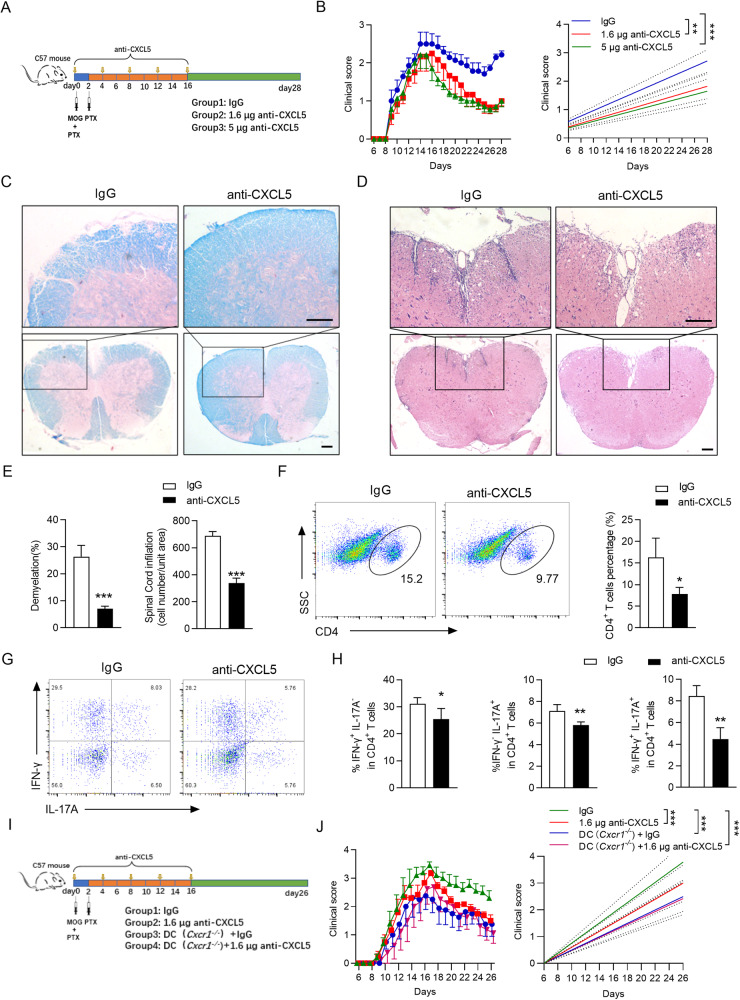
Inhibition of CXCL5 action attenuates EAE progression. **A** Schematic diagram of EAE mice treated with CXCL5 neutralizing antibody (anti-CXCL5) or isotype control (IgG). **B** Clinical scores (left panel) and linear regression analysis (right panel) of the recipient mice. The data are expressed as the mean ± SEM (*n* = 7). ^**^p < 0.01, ^***^p < 0.001 vs. IgG group (Mann–Whitney *U* test). **C** LFB staining and **D** H&E staining of spinal cord paraffin sections from the IgG and anti-CXCL5 (5 μg) treated mice 28 days after EAE induction. Pooled data are presented in the right panel. **E** Pooled data are presented from (**C**, **D**). **F** Frequencies of CD4^+^ T cells and **G** expression of IFN-γ and IL-17A from the spinal cord and brain 28 days after EAE induction. **H** Pooled data are presented from (**G**). ^*^p < 0.05, ^**^p < 0.01, ^***^p < 0.001 vs. IgG group (one-tailed Student’s *t*-test). **I** Schematic diagram of DC (WT) or DC (*Cxcr1*^−/−^) mice that were induced EAE and treated with 1.6 μg anti-CXCL5 or IgG. **J** Clinical scores (left panel) and linear regression analysis (right panel) of the recipient mice. The data are expressed as the mean ± SEM (*n* = 7). ^**^p < 0.01, ^***^p < 0.001 vs. IgG group (Mann–Whitney *U* test).

To unequivocally demonstrate the participation of CXCL5 in disease progression, we first analyzed the expression of CXCL5 in serum and the mRNA level of *Cxcl5* in the brain of the EAE model. Of note, we found a significant upregulation of CXCL5 expression in serum in the early period of EAE progression and *Cxcl5* mRNA in the brain in the later period of EAE progression (Supplementary information, Fig. S9A, B). An increased expression of CXCL5 both in protein and mRNA suggests that CXCL5 at least participates in the progression of EAE. Next, we neutralized CXCL5 to analyze the function of CXCL5 in EAE pathogenesis. C57BL/6 mice were induced EAE animal model by MOG_35–55_ and were treated at day 0/4/8/12/16 post-immunization with intraperitoneal injection of neutralizing anti-CXCL5 antibody or control IgG (Fig. 4A). After anti-CXCL5 antibody treatment, lower clinical scores were observed in mice received 1.6 μg or 5 μg anti-CXCL5 antibody compared to mice injected with IgG (Fig. 4B), with lower associated pathology (leukocyte infiltration, inflammatory foci, and demyelination) (Fig. 4C–E). CD4^+^ T cells isolated from CNS were quantified, and decreased percentages of CD4^+^ T cells were found in CXCL5 neutralized mice compared to those in IgG-treated mice (Fig. 4F). The number of Th1 and Th17 cells in the CNS showed a significant decrease in 5 μg anti-CXCL5 antibody-treated mice (Fig. 4G, H). To determine whether CXCL5 is a receptor agonist for CXCR1 to mobilize the production of inflammatory cytokines in DCs, we first measured the expression of CXCL5 in vitro. We observed the high level of CXCL5 in DCs with LPS stimulation was consistent with DCs co-cultured with naïve CD4^+^ T cells (Supplementary information, Fig. S9C, D). We next assessed the impact of CXCL5 in DC (*Cxcr1*^−/−^) mice during the progression of EAE. Both DC (*Cxcr1*^−/−^) mice and DC (*Cxcr1*^−/−^) mice with anti-CXCL5 antibody treatment reduced the clinical score of EAE. Meanwhile, there were no significant different between DC (*Cxcr1*^−/−^) mice and DC (*Cxcr1*^−/−^) mice with anti-CXCL5 antibody treatment (Fig. 4I, J). Overall, the data demonstrated that CXCL5 aggressively participates in the progression of EAE through CXCR1.

### HIF-1α is necessary for CXCL5/CXCR1 axis-mediated proinflammatory cytokine production in DCs

**Fig. 5:**
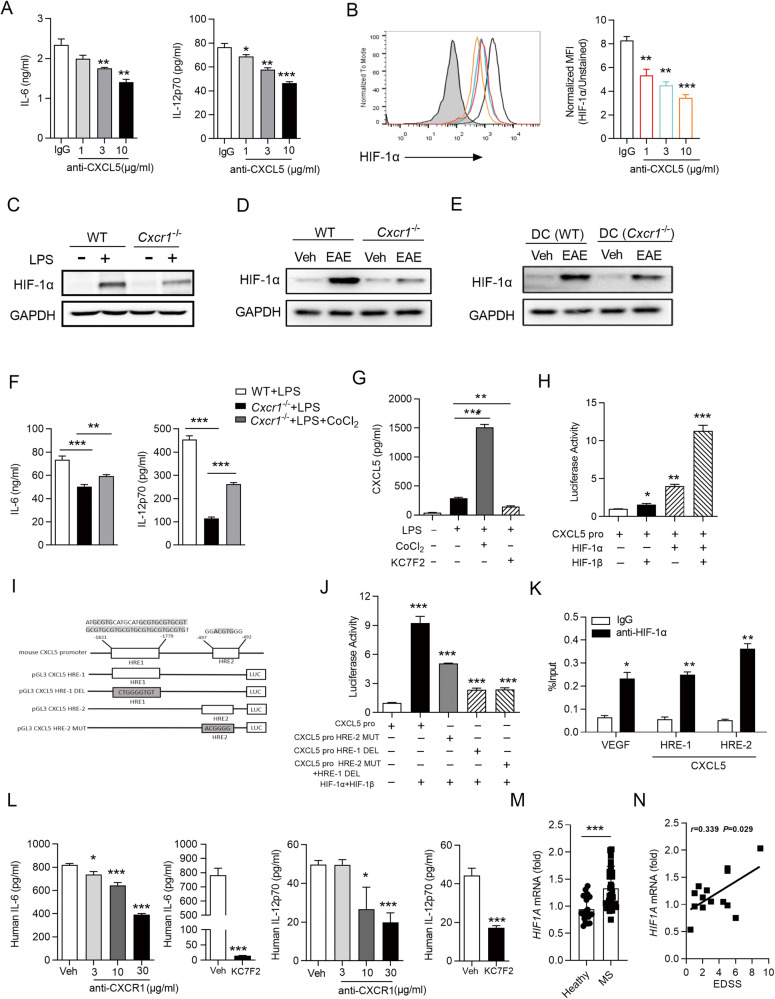
CXCL5/CXCR1/HIF-1α feedback loop positively regulates the production of IL-6 and IL-12p70 in DCs. DCs were sorted from spleens of WT mice and treated with LPS (100 ng/ml) for 24 h in the presence of anti-CXCL5 antibody or IgG isotype control. **A** Concentrations of IL-6 and IL-12p70 in culture supernatants of DCs were detected by ELISA. **B** Flow cytometry analyzed the expression of HIF-1α (left), Pooled data are presented from (right). **C** DCs were sorted from spleens of WT or *Cxcr1*^−/−^ mice and stimulated by LPS or not for 24 h. The expression of HIF-1α was examined by Immunoblot analysis. **D**, **E** Four different groups of mice (WT, *Cxcr1*^−/−^, DC(WT) and DC(*Cxcr1*^−/−^)) were immunized with MOG _35–55_ peptide in CFA adjuvant and pertussis toxin to induce EAE for 28 days. The spinal cord and brain lysates were probed for HIF-1α protein level by Immunoblot analysis. **F** Purified DCs were sorted from spleens of WT or *Cxcr1*^−/−^ mice and treated for 24 h with LPS (100 ng/ml) in the presence or absence of the HIF-1α agonist (CoCl_2_, 200 μM). Concentrations of IL-6 and IL-12p70 in supernatants were detected by ELISA. **G** Purified DCs were treated for 24 h with LPS (100 ng/ml) in the presence or absence of the HIF-1α agonist (CoCl_2_, 200 μM) or inhibitor (KC7F2, 20 μM) and concentrations of CXCL5 in supernatants were detected by ELISA. **I** Schematic diagram of two HREs in the CXCL5 promoter. **H**, **J** Luciferase assay to analyze the function of HIF-1α in regulation of CXCL5 promoter activity. Luciferase activity was calculated as the ratio of firefly/Renilla luciferase activity. **K** ChIP assay demonstrating the direct binding of HIF-1α to CXCL5 promoter. **L** IL-6 or IL-12p70 production by human DCs stimulated with LPS (100 ng/ml) alone or combined with indicated conditions. **M** *HIF1A* mRNA level in peripheral blood leukocytes from MS patients (*n* = 20) or control healthy donors (*n* = 18). **N** Scatterplots showing the correlation between mRNA level of *HIF1A* and EDSS. Data are mean ± SEM. ^*^p < 0.05, ^**^p < 0.01, ^***^p < 0.001 vs. WT or indicated group (one-tailed Student’s *t*-test). Data are representative of three independent experiments with similar results.

To further evaluate the effect of CXCL5 in DCs-mediated proinflammatory cytokine production, we first sorted the DCs from the spleens of wild-type and then stimulated DCs with LPS under CXCL5 neutralization antibody and found that CXCL5 neutralization led to significantly decreased IL-6 and IL-12p70 production by DCs (Fig. 5A). To determine specific molecular pathways, DCs were isolated from *Cxcr1*^−/−^ and WT mice to for further detection of phosphorylation on extracellular signal-regulated kinases (ERK), protein kinase B (AKT), S6, p38, and c-Jun N-terminal kinase (JNK) after stimulated with LPS. *Cxcr1* deficiency leads to significantly reduced phosphorylation of ERK, not the phosphorylation of AKT, S6, p38, and JNK (Supplementary information, Fig. S10A). HIF-1α has been reported as being activated by phosphorylated ERK and implicated in controlling cytokine production such as IL-6, and IL-12p70 [41, 42], we investigated the effect of inhibiting ERK phosphorylation on activation of HIF-1α in DCs. FACS and Immunoblot analysis showed that ERK inhibitor (U0126) treatment significantly decreased the expression of HIF-1α in DCs (Supplementary information, Fig. S10B). To further investigate if HIF-1α regulates the IL-6 and IL-12p70 production by DCs, we inhibited HIF-1α with a hypoxic inhibitor (KC7F2) and found KC7F2 treatment led to significantly decreased production of IL-6 and IL-12p70 in DCs (Supplementary information, Fig. S11). Then we interrogated whether HIF-1α expression was affected by CXCL5/CXCR1 axis in DCs and found CXCL5 neutralization and *Cxcr1* deficiency both greatly reduced the protein level of HIF-1α in DCs (Fig. 5B, C) (Supplementary information, Fig. S12). We next tested the protein level of HIF-1α during the progression of EAE, spinal cords were isolated from WT and *Cxcr1*^−/−^ mice at 0 day and 26 days after EAE induction; the data showed that *Cxcr1* deficiency leads to prominently decreased HIF-1α expression at 26 days after EAE induction (Fig. 5D), consistent with the tendency of HIF-1α in DC (*Cxcr1*^−/−^) mice (Fig. 5E). We further analyzed whether HIF-1α was required to mediate the regulation function of CXCR1 on IL-6 and IL-12p70 production in DCs, results from cytokine detection revealed that HIF-1α agonist (CoCl_2_) significantly retuned decreased expressions of IL-6 and IL-12p70 caused by CXCR1 deficiency in DCs (Fig. 5F). These results demonstrated that CXCL5/CXCR1 positively regulates inflammation secretion function of DCs via HIF-1α.

### CXCL5/CXCR1/HIF-1α feedback loop positively regulates IL-6 and IL-12p70 secretion in DCs

It is known that HIF-1α is a transcription factor with the ability to regulate its own upstream activators [43, 44]. We, therefore, explored the possibility that HIF-1α upregulates CXCL5 expression, thereby forming a positive feedback loop to control inflammatory cytokines production in DCs. Interestingly we really found that the production of CXCL5 in DCs was greatly enhanced upon HIF-1α activator treatment, while significantly decreased by HIF-1α inhibitor treatment (Fig. 5G). We speculate that HIF-1α may regulate CXCL5 transcription by direct binding to its promoter region, data from luciferase reporter assay showed that luciferase activity of the *Cxcl5* promoter can be significantly enhanced by overexpression of HIF-1α and 1β (Fig. 5H). Combined with the fact that two putative hypoxia response elements (HREs) containing the consensus sequence (A/G)CGTG were found within the promoter of mouse CXCL5 by using the JASPAR database (Fig. 5I), while the enhanced luciferase activity by overexpression of HIF was greatly impaired when we mutated the HIF binding sites in the promoter region of *Cxcl5* (Fig. 5J). Moreover, data from chromatin immunoprecipitation (ChIP) analyses further confirmed the direct binding of HIF-1α onto the promoter region of *Cxcl5* (Fig. 5K). These observations confirmed that HIF-1α is a direct transcriptional factor for CXCL5.

Further, we confirmed the roles of CXCR1 and HIF-1α regulating of IL-6 and IL-12p70 secretion in human DCs. We found that inhibition of any of CXCR1 or HIF-1α can lead to a significant reduction of IL-6 and IL-12p70 production in human DCs (Fig. 5L). Moreover, we also examined the mRNA level of *HIF1A* in peripheral blood leukocytes of healthy control and MS patients and found a significant increase of *HIF1A* in MS patients (Fig. 5M) and a positive correlation between *HIF1A* expression level and EDSS scores (Fig. 5N). These results confirmed that CXCR1 governs IL-6 and IL-12p70 production via increasing CXCL5/CXCR1/HIF-1α positive feedback loop in DCs.

### Deletion of CXCR1 prevents LPS-induced ARDS

**Fig. 6:**
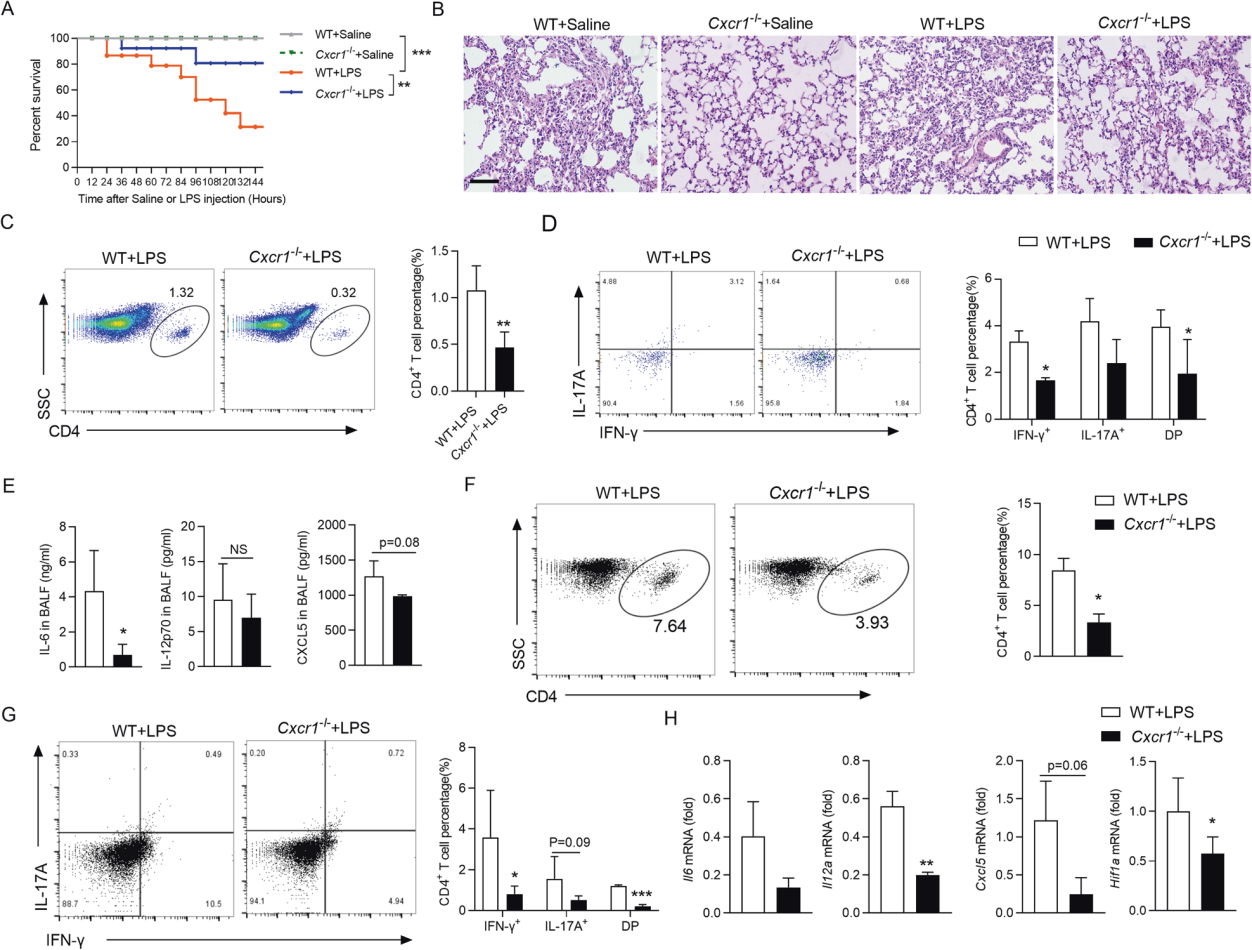
Deletion of CXCR1 protects from LPS-induced acute lung injury. **A** Percent survival of WT and *Cxcr1*^−/−^ mice after LPS (10 mg/kg) intraperitoneal compared with saline controls. (*n* = 8, Kaplan–Meier survival curves/Mantel–Cox analysis). **B** H&E staining images of lung tissue from WT and *Cxcr1*^−/−^ mice after saline or 10 mg/kg LPS challenge. Tissue was collected 24 h after administration. **C**–**E** Bronchoalveolar lavage fluid (BALF) was obtained after WT and *Cxcr1*^−/−^ mice 24 h intraperitoneal injection of LPS (10 mg/kg). Representative flow cytometry data showing frequencies of CD4^+^ T cells (**C**) IFN-γ^+^ and IL-17A^+^ (**D**). Pooled data are presented in the right panel. **E** ELISA analysis of IL-6, IL-12p70, and CXCL5 concentrations in BALF. **F**, **G** Immune cells were sorted from the lung tissue, and the percentage of CD4^+^ T cells (**F**), IFN-γ, and IL-17A in CD4^+^ T (**G**) were analyzed by flow cytometry. Pooled data are presented in the right panel. **H** Quantitation of *Il6*, *Il12a*, *Cxcl5*, and *Hif1α* mRNA level in lung tissue of WT and *Cxcr1*^−/−^ mice stimulated with LPS for 24 h. Data are mean ± SEM (*n* = 5–6 in **B**–**E**, **G**, **H**). ^*^p < 0.05, ^**^p < 0.01, ^***^p < 0.001 vs. WT group (one-tailed Student’s *t*-test). Data are representative of three independent experiments with similar results.

As DCs-mediated IL-6 production also plays a pivotal role in the pathogenesis of LPS-induced ARDS [45, 46], we further examined whether the CXCL5/CXCR1 axis is necessary for IL-6 production in LPS-induced ARDS. Compared with the WT mice, the level of CXCL5 in serum and BALF increased when stimulated with LPS (Supplementary information, Fig. S13A, B). Thus, we further examined whether CXCR1 deficiency could ameliorate ARDS. By i.p. injected with a single bolus of 10 mg/kg LPS, the mouse ARDS model was induced, and a 6-day survival rate was calculated. The results indicated that *Cxcr1*^−/−^ mice showed a significantly higher survival rate compared to WT mice (Fig. 6A). Histological assessment of lung sections showed that CXCR1 deficiency prevented septa thickening and inhibited infiltration of inflammatory cells into the bronchioles (Fig. 6B). Importantly, *Cxcr1*^−/−^ mice showed a strongly decreased total CD4^+^ T cells and IFN-γ^+^ and IL-17A^+^ percentage in CD4^+^ T cells (Fig. 6C, D), and accompanied by decreased IL-6 and CXCL5 in BALF (Fig. 6E). Consistently, mice lacking CXCR1 were protected from LPS-induced ARDS by exhibiting reduced CD4^+^ T cell percentage (Fig. 6F), the proportion of IFN-γ^+^ and IL-17A^+^ (Fig. 6G) and the mRNA levels of *Il6*, *Il12a*, *Cxcl5*, and *Hif1α* in lung tissue (Fig. 6H). Flow cytometry analysis and western blot suggested that CXCR1 deficiency resulted in the loss of HIF-1α protein level in lung tissue (Supplementary information, Fig. S13C, D).

**Fig. 7:**
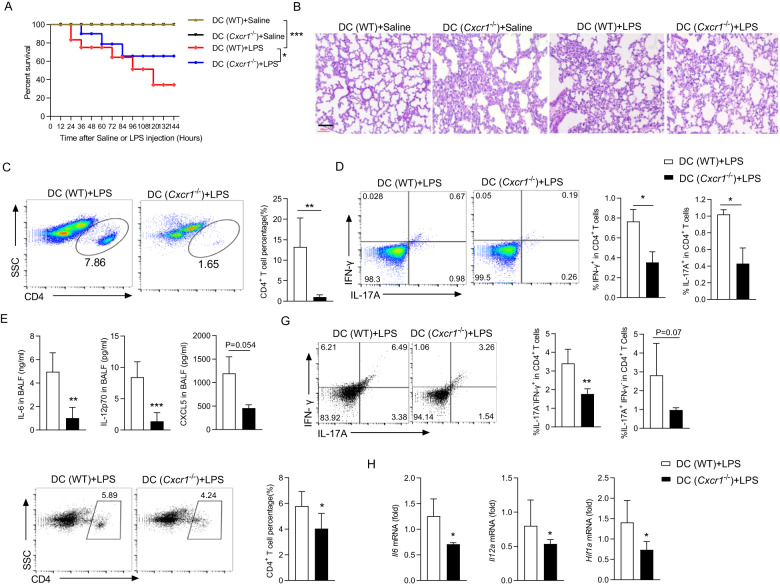
CXCR1 governs the pathogenesis of LPS-induced acute lung injury in DCs. **A** Percent survival of DC(WT) and DC(*Cxcr1*^−/−^) mice after LPS (10 mg/kg) intraperitoneal compared with saline controls. (*n* = 8, Kaplan–Meier survival curves/Mantel–Cox analysis). **B** H&E staining images of lung tissue from DC(WT) and DC(*Cxcr1*^−/−^) mice after saline or 10 mg/kg LPS challenge. Tissue was collected 24 h after administration. **C**–**E** Bronchoalveolar lavage fluid (BALF) was obtained after DC(WT) and DC(*Cxcr1*^−/−^) mice 24 h intraperitoneal injection of LPS (10 mg/kg). Representative flow cytometry data showing frequencies of CD4^+^ T cells (**C)** IFN-γ^+^ and IL-17A^+^ (**D**). Pooled data are presented in the right panel. **E** ELISA analysis of IL-6, IL-12p70, and CXCL5 concentrations in BALF. **F**, **G** Immune cells were sorted from the lung tissue, and the percentage of CD4^+^ T cells (**F**) and the proportion of IFN-γ and IL-17A in CD4^+^ T cells (**G**) were analyzed by flow cytometry. Pooled data are presented in the right panel. **H** Quantitation of *Il6*, *Il12a*, and *Hif1α* mRNA levels in lung tissue of DC(WT) and DC(*Cxcr1*^−/−^) mice stimulated with LPS for 24 h. Data are mean ± SEM (*n* = 5–6 in **B**–**E**, **G**, **H**). ^*^p < 0.05, ^**^p < 0.01, ^***^p < 0.001 vs. DC (WT) grou*p* (one-tailed Student’s *t*-test). Data are representative of three independent experiments with similar results.

Next, we tested whether CXCR1 deficiency in DCs can ameliorate LPS-induced ARDS. As expected, DC(*Cxcr1*^−/−^) mice exhibited higher survival rate than DC(WT) mice after LPS i.p. injection (Fig. 7A). Histological pathogenesis of lung sections, cytokine production, IFN-γ^+^ and IL-17A^+^ percentage of CD4^+^ T cells in BALF were all significantly alleviated in mice with conditional deletion of *Cxcr1* in DCs (Fig. 7B–E). In agreement with the data from *Cxcr1*^−/−^ mice, DC(*Cxcr1*^−/−^) mice showed reduced CD4^+^ IFN-γ^+^ T and CD4^+^ IL-17A^+^ T cell percentage (Fig. 7F, G) and the decreased mRNA levels of *Il6*, *Il12a*, and *Hif1α* in lung tissue compared with the DC(WT) mice (Fig. 7H). We concluded that CXCR1 deficiency can ameliorate LPS-induced ARDS, and the CXCL5/CXCR1/HIF-1α positive feedback loop also inhibits the production of inflammatory cytokines in LPS-ARDS.

## Discussion

**Fig. 8:**
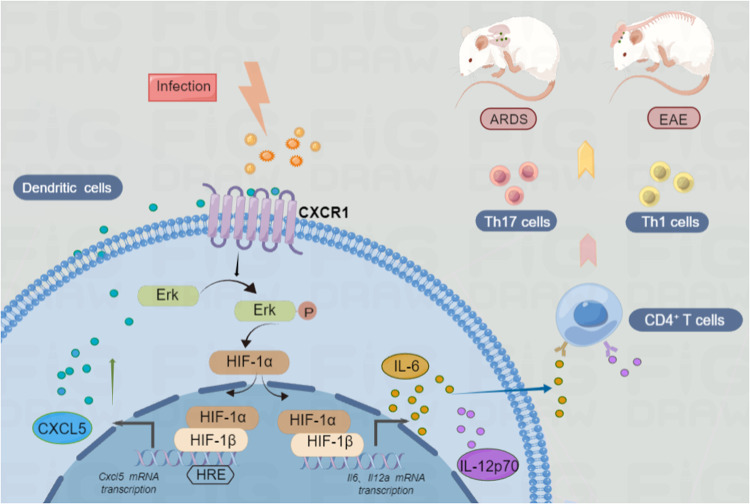
Graphical summary of CXCR1 drives dendritic cell-dependent inflammation and autoimmune pathogenesis. LPS or virus infection upregulates the level of HIF-1α via CXCR1 and Erk signaling pathways in DCs. The increasing level of HIF-1α binds to the CXCL5 promoter and causes an upregulation of CXCL5 expression, then forming a CXCL5/CXCR1/HIF-1α positive feedback loop. This feedback loop promotes IL-6 and IL-12p70 expression, ultimately leading to inflammation damage in the EAE and ARDS mice model. LPS lipopolysaccharide, DC dendritic cells, ARDS acute respiratory distress syndrome.

DCs are critical to driving the development of a myriad of autoimmune and inflammatory diseases because of their antigen-presenting function and high expression of anti-inflammation and pro-inflammation cytokines [47, 48]. It is, therefore, important to understand the mechanisms controlling cytokines secretion in DCs. In this study, we identified a mechanism that positively regulates DCs function through CXCR1 and its CXCL5 in mice. We showed that CXCR1 strengthened the pro-inflammation cytokines production of DCs and further accelerated inflammation and autoimmunity disease progression via a positive feedback loop of CXCL5/CXCR1/HIF-1α (Fig. 8).

Previous studies have shown that IL-6 and IL-12p70 secreted by DCs induce naïve CD4^+^ T cells to Th17 and Th1 differentiation. Most studies about the function of CXCR1 were focused on neutrophils and DCs, such as degranulation and migration, while its influence on the pro-inflammation production of DCs has not been fully understood [49, 50]. In this study, we found that CXCR1 in DCs promoted the expression of IL-6 and IL-12p70; however, there was no change in the expression of TGF-β1 in vitro and in vivo. This suggested that CXCR1 in DCs specifically regulated IL-6 and IL-12p70. Consistent with these findings, neutralized CXCL5, the expression of IL-6 and IL-12p70 also decreased. Multiple mechanisms may contribute to the inflammation cytokine secretion of DCs during autoimmunity, and mitogen-activated protein kinase (MAPK) has been shown to play a significant role in positive modulation of the inflammation cytokine secretion [51, 52]. Indeed, we found that CXCR1 altered ERK phosphorylation but not p38, PI3K-AKT, or JNK signaling pathways in DCs.

Previous studies have revealed that CXCR1 and CXCR2 receptors share 76% sequence homology with each other and interact with ELR^+^ CXCL chemokines playing high significance in many inflammatory diseases [49]. However, differences in the affinities of CXCR1 and CXCR2 towards CXCL chemokines monomer versus dimer may lead to different signaling outcomes, such as phospholipase D (PLD) activation and superoxide anion production in neutrophils is exclusively mediated by CXCR1, but not CXCR2 [53]. In autoimmune diseases, CXCL3-induced migration becomes exclusively CXCR1-dependent, which underlines the significance of CXCR1 in the pathogenesis of airway remodeling associated with asthma [54]. Our data indicated that *CXCR1* mRNA in the peripheral white blood cells of MS patients increased more than compared with healthy controls. Further investigation is therefore warranted to understand how CXCR1 contributes to disease progression during inflammation and autoimmunity.

As a master transcription factor, HIF-1α is situated at the convergence of multiple oncogenic and tumor suppressor pathways, including MAPK/ERK pathways [42]. A previous study found that HIF-1α promoted the production of inflammatory cytokines from macrophages, including IL-6, IL-12p70, and IL-1 in LPS-induced sepsis. This result was in congruence with our results that showed inhibiting HIF-1α could downregulate IL-6 and IL-12p70 in LPS-induced DCs. We also discovered that HIF-1α promoted CXCL5 expression by directly activating CXCL5 transcription. Meanwhile, our study went one step forward by revealing that once HIF-1α was triggered by CXCR1, the transcription of CXCL5 continued to elevate, suggesting that a positive feedback loop containing CXCR1 and HIF-1α is involved in the transcription of CXCL5.

## Conclusion

Collectively, this study demonstrated that pro-inflammation secretion of DCs driven by the CXCR1 played an inflammation-promoting role in inflammation and autoimmunity diseases, and a mechanism was further revealed whereby CXCR1 promoted IL-6 and IL-12p70 production via CXCL5/CXCR1/HIF-1α positively feedback loop. Given the important role of CXCR1 in LPS-infected ARDS development and progression, we propose that CXCR1 may be a future therapeutic approach in such diseases.

## Supplementary information

supplement figure

Original Data File

Reproducibility Checklist

## Data Availability

All datasets generated and analyzed during this study are included in this published article and its Supplementary Information files. Additional data are available from the corresponding author upon reasonable request.
