# Supplementary material for: CXCR1 drives the pathogenesis of EAE and ARDS via boosting dendritic cells-dependent inflammation
Source: Cell Death Dis. 2023 Sep 14;14(9):608. doi: 10.1038/s41419-023-06126-y (PMC10502121; doi:10.1038/s41419-023-06126-y)
Supplement: Supplementary file 1 — supplement figure [file 41419_2023_6126_MOESM1_ESM.docx]

**Table S1. Primer sequences used in this study**

| Gene | Forward (5’-3’) | Reverse (5’-3’) |
| --- | --- | --- |
| Human*CXCR1* | TGAGCCCCGAATCTGACATT | CAGCTGGCAGGTTGATGTTT |
| Human*HIF1A* | GAACGTCGAAAAGAAAAGTCTCG | CCTTATCAAGATGCGAACTCACA |
| Human*GAPDH* | GCTGTGGGCAAGGTCATCC | GCCTGCTTCACCACCTTCTT |
| Mouse*Cxcr1* | AGCCCACTTGATTGAAGATACT | CCAACGAAGGCATAGATGATAG |
| Mouse*Cxcl5* | GAAAGCTAAGCGGAATGCAC | GGGACAATGGTTTCCCTTTT |
| Mouse*Il6* | TACCACTTCACAAGTCGGAGGC | CTGCAAGTGCATCATCGTTGTTC |
| Mouse*Il12a* | CCCTGTGCCTTGGTAGCATC | CCTTAGTGTTGATAGCAATGGTGA |
| Mouse*Tgfb1* | CACTGATACGCCTGAGTG | GTGTGCGCTGAATCGAAA |
| Mouse*Hif1α* | GAATGAAGTGCACCCTAACAAG | GAGGAATGGGTTCACAAATCAG |
| Mouse*Gapdh* | CGTGCCGCCTGGAGAAACCTG | GAGTGGGAGTTGCTGTTGAAGTCGC |

**
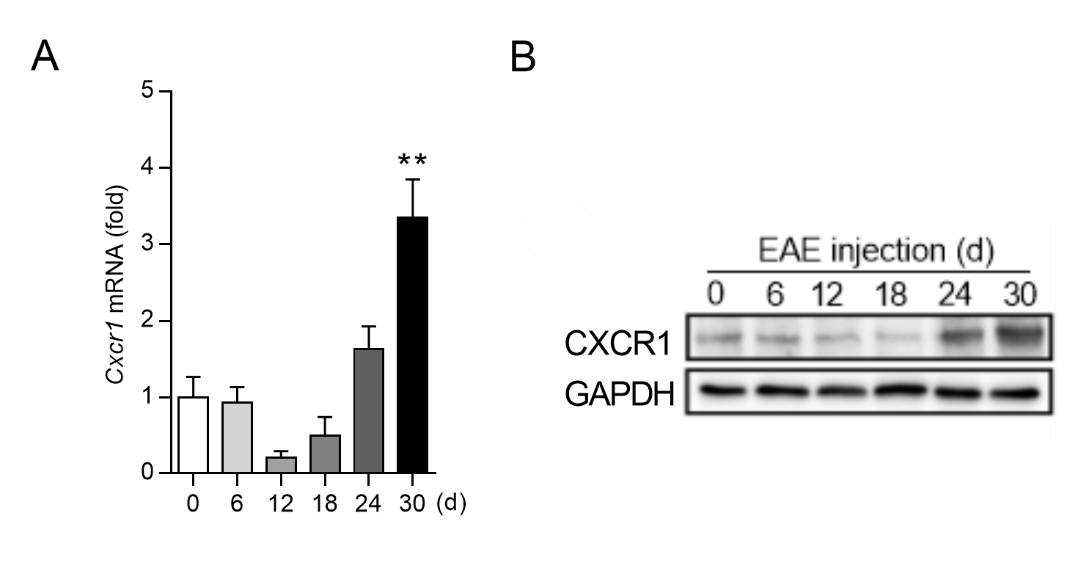
**

**Figure S1. CXCR1 expression in CNS of EAE mice.**


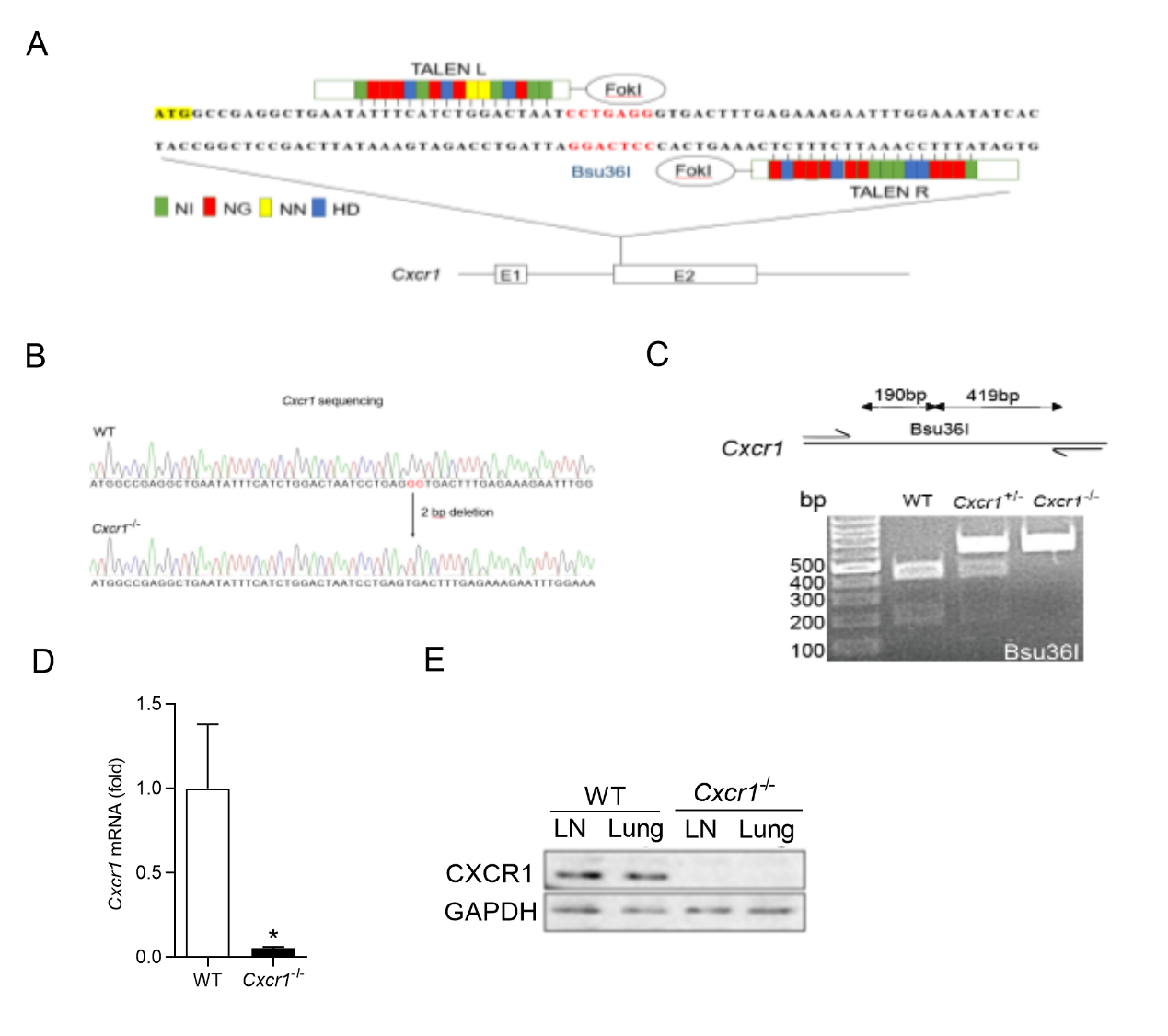
(A) *Cxcr1* mRNA expression from the CNS of WT after EAE induction for specified time was detected by RT-qPCR. (B)Western blot assayed the protein level of CXCR1 from the CNS of WT after EAE induction for specified time. Data are representative of two independent experiments (mean ± SEM; n=5-7). ^**^*p*<0.01.

**Figure S2. *Cxcr1*^−/−^ mice were generated.**

(A) TALEN-binding sites with Cxcr1 exon 2 and start codon (yellow) in the protein coding sequence. (B) DNA sequencing reads of a WT and *Cxcr1*^-/-^ allele from the founder F0 mouse. A deletion of 2bp (GG) in the *Cxcr1*^-/-^ allele. (C) Genotyping of Cxcr1 knockout mice by PCR and endonuclease Bsu36I digestion. (D) The mRNA expression of *Cxcr1* in WT and *Cxcr1*^-/-^ mice. (E) Western blot detected the CXCR1 protein expression in lymph nodes and lung. Data are mean ± SEM (n=5-6). **p*<0.05, one-tailed Student’s *t*-test. Data were from one representative of three independent experiments.


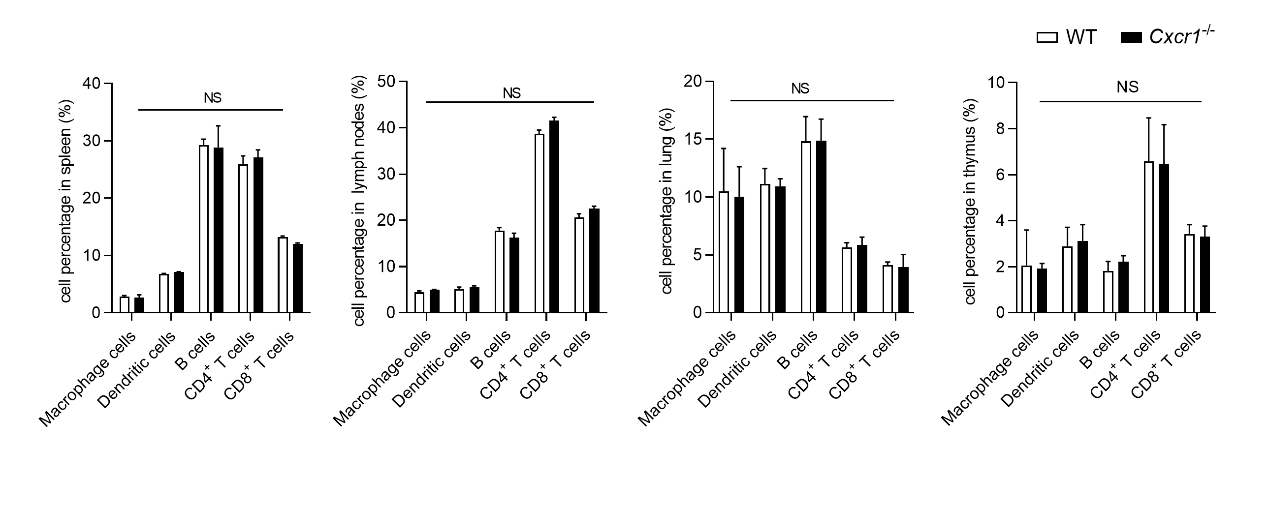


**Figure S3. CXCR1 deficiency did not mediate developmental defects of immune cells *in vivo*.** Percentages of the indicated immune cells were analyzed by flow cytometry from WT and *Cxcr1*-deficient mice. Data are mean ± SEM (n=5-6). one-tailed Student’s *t*-test. Data were from one representative of three independent experiments.


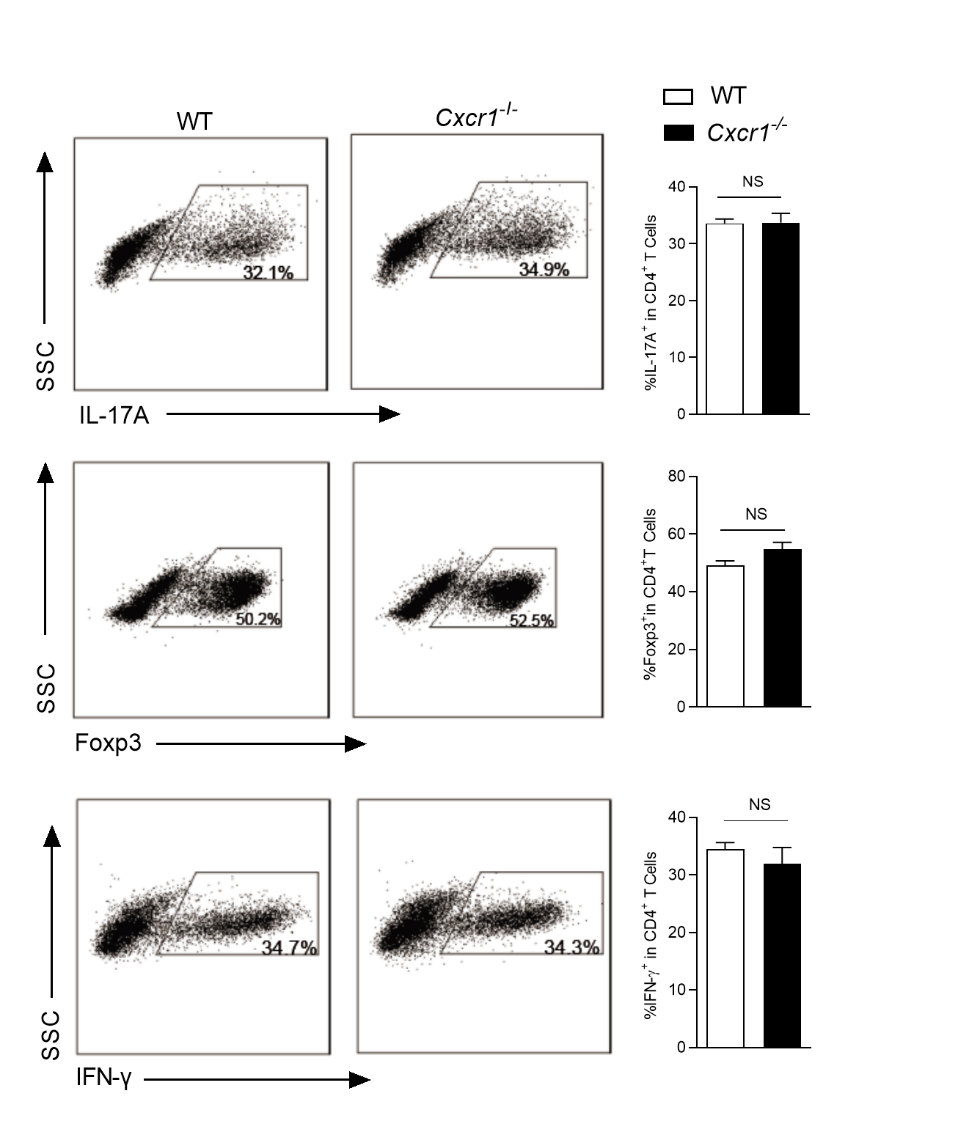


**Figure S4. CXCR1 did not affect T cell differentiation directly *in vitro*.** Naive CD4^+^ T cells isolated from the spleen of 6–8-wk-old wild-type mice and *Cxcr1^-/-^* mice were induced to differentiate into Th1 cells, Th17 cells, or Treg cells *in vitro*. The percentage of Th1 cells, Th17 cells, and Treg cells were analyzed by FACS with intracellular staining of IFN-γ, IL-17A, or Foxp3, respectively. Data are mean ± SEM (n=5-6). one-tailed Student’s *t*-test. Data were from one representative of three independent experiments.


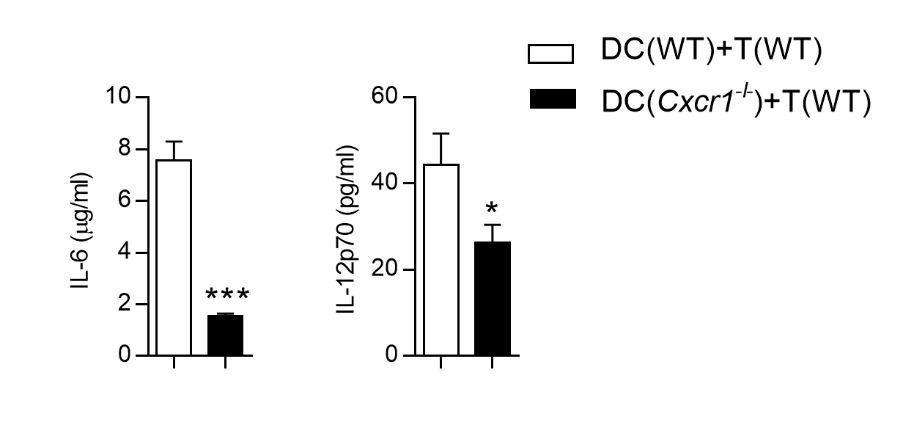


**Figure S5. CXCR1 deficiency in DCs leads to reducing IL-6 and IL-12p70 production.** DCs were sorted from spleens of DC (WT) or DC (*Cxcr1*^-/-^) mice and treated with LPS (100 ng/ml) for 24 hr. Activated DCs were co-cultured with T cells from spleens of WT for 3 days. Concentrations of IL-6 and IL-12p70 in supernatants were detected by ELISA. Data are mean ± SEM (n=5-6). ^*^*p*<0.05, ^***^*p*<0.001 versus WT mice group (one-tailed Student’s *t*-test). Data are representative of three independent experiments with similar results.


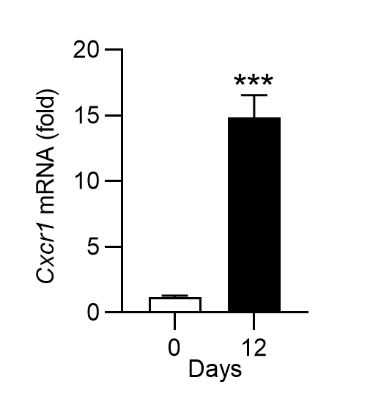


**Figure S6. *Cxcr1* expression in DCs from the spleen of EAE mice.** DCs were sorted by using MagniSort® Mouse CD11c Positive Selection Kit from the spleen of WT mice and WT mice after EAE induction for 12 days. *Cxcr1* mRNA expression was detected by RT-qPCR. Data are representative of two independent experiments (mean ± SEM; n=5-7). ^***^*p*<0.001.

**
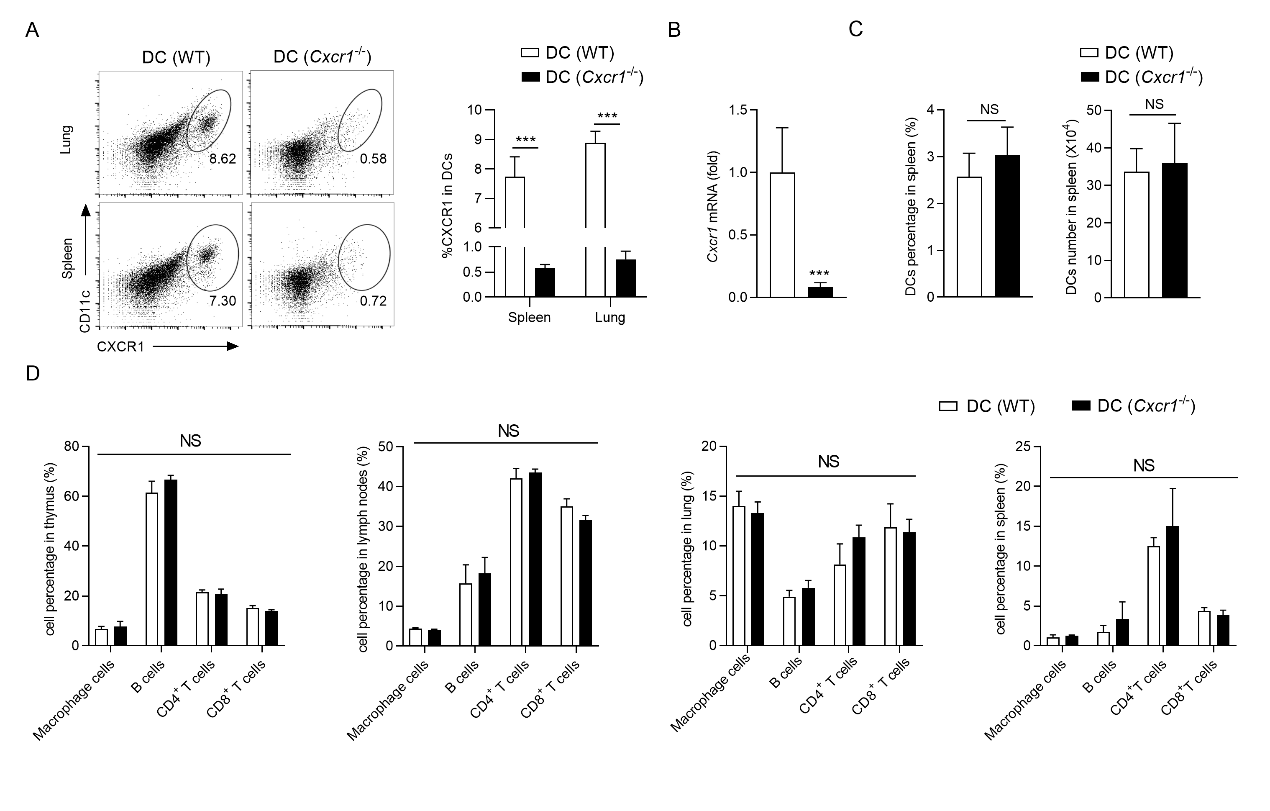
**

**Figure S7. CXCR1 deficiency in DCs does not mediate developmental defects of immune cells *in vivo*.** (A) DCs were sorted from spleen and lung of DC (WT) an DC (*Cxcr1*^-/-^) mice and percentages of CXCR1 was analyzed by flow cytometry. (B) DCs were sorted from spleen of DC (WT) and DC (*Cxcr1*^-/-^) mice, after cultured for 7 days *in vitro*. The mRNA level of *Cxcr1* was detected by RT-qPCR. (C) Spleen was collected from DC (WT) or DC (*Cxcr1*^-/-^) mice. Percentage and total number of DCs in splenocytes were analyzed by flow cytometry. (D) Percentages of the indicated immune cells were analyzed by flow cytometry from WT and CXCR1-deficient mice. Data are mean ± SEM (n=5-6). ****p*<0.001, one-tailed Student’s *t*-test. Data were from one representative of three independent experiments.


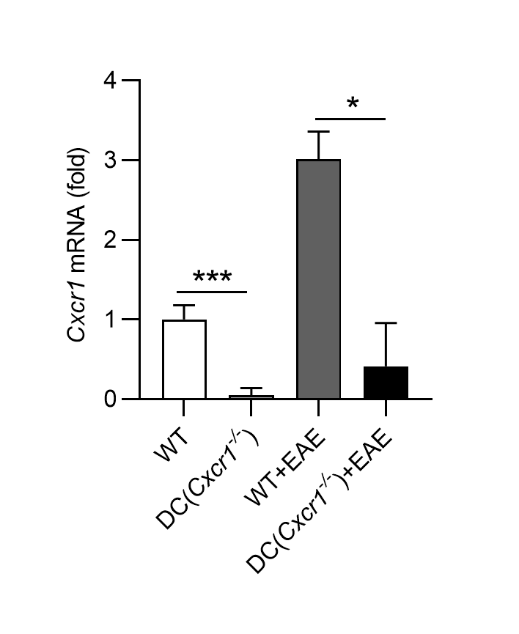


**Figure S8. *Cxcr1* expression in brain of DC (WT) an DC (*Cxcr1^-/-^*) mice with EAE.**

DC (WT) an DC (*Cxcr1^-/^*^-^) mice were induced EAE, and the brain were collected at 26 days after EAE induction. *Cxcr1* mRNA expression was detected by RT-qPCR. Data are representative of two independent experiments (mean ± SEM; n=5-7). ^*^*p*<0.05. ****p*<0.001. one-tailed Student’s *t*-test. Data were from one representative of three independent experiments.


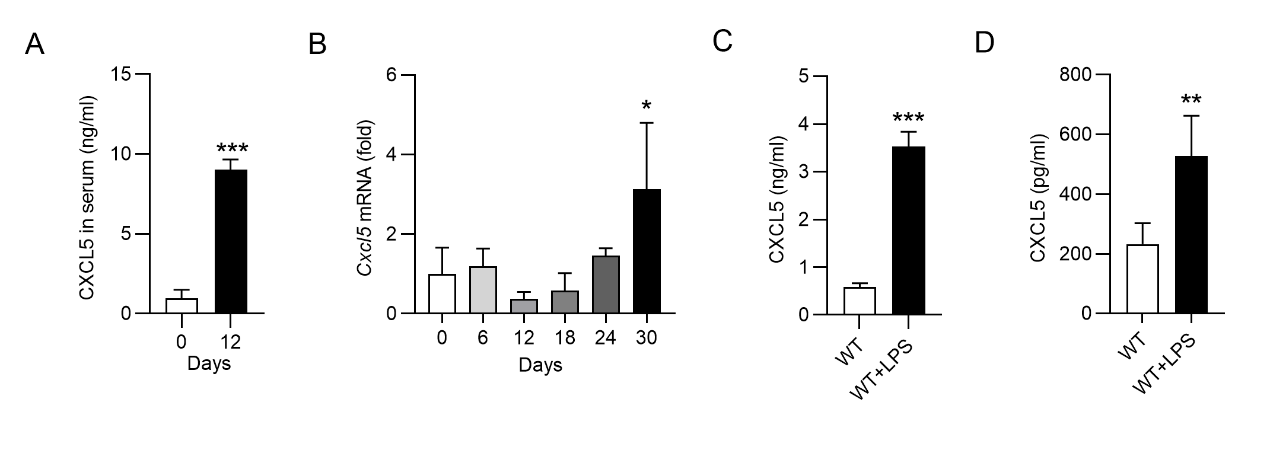


**Figure S9. CXCL5 expression in EAE mice and *in vitro*.** (A) The expression of CXCL5 in serum from WT after EAE induction for 12 days were detected by ELISA. (B) RT-qPCR assayed *Cxcl5* mRNA level from the brain of WT after EAE induction for specified time. (C) DCs were sorted from bone marrow and cultured for 7 days. After LPS stimulated for 24 hr, the expression of CXCL5 in supernatant was detected by ELISA. (D) DCs were sorted from bone marrow and cultured for 7 days. After LPS stimulated for 24 hr, DCs and T cells were co-cultured for 3 days. The expression of CXCL5 in supernatant was detected by ELISA. (mean ± SEM; n=5-7). ^*^*p*<0.05, ***p*<0.01, ****p*<0.001. one-tailed Student’s *t*-test. Data were from one representative of three independent experiments.


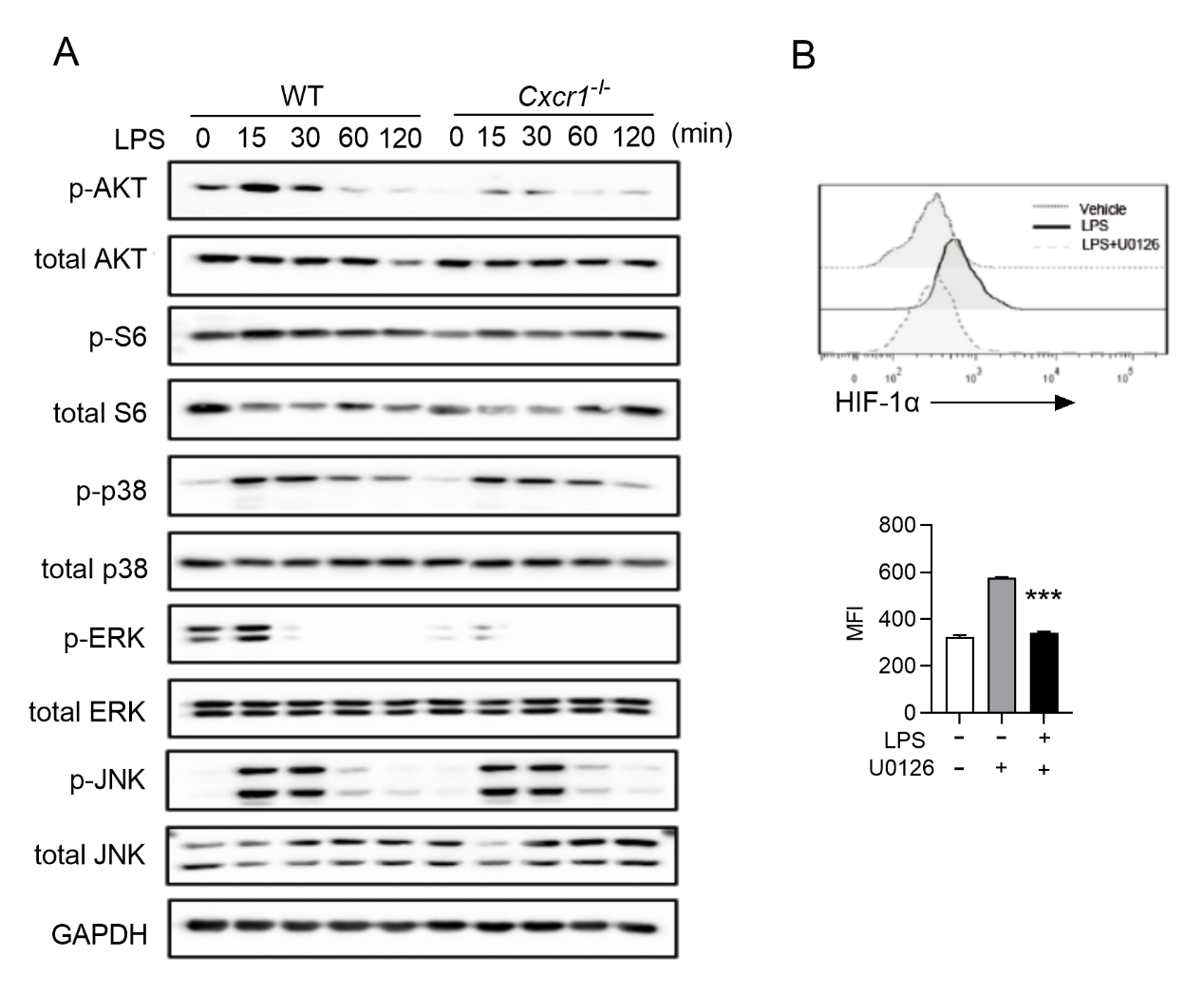


**Figure S10. CXCR1 positively regulated HIF-1α via ERK signaling pathway in DCs.** (A) DCs were sorted from spleen of WT or *Cxcr1*^-/-^ mice and were treated with LPS (100 ng/ml) for specified time. DC lysates were probed for phosphorylated or total ERK, AKT, S6, p38 and JNK. (B) DCs were sorted from spleen of WT mice and were treated with LPS (100 ng/ml) and combined with ERK inhibitor (U0126) or not, protein level of HIF-1α was analyzed by flow cytometry. Data are mean ± SEM (n=5-6). ^***^*p*<0.001 versus WT mice group (one-tailed Student’s *t*-test). Data are representative of three independent experiments with similar results.


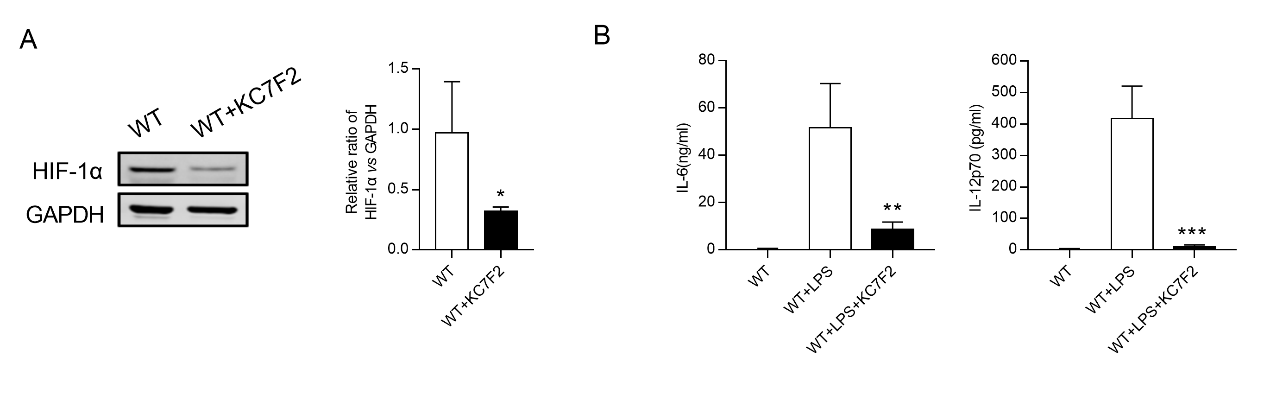


**Figure S11. Production of IL-6 and IL-12p70 are dependent on HIF-1α in DCs activated by LPS.** DCs were sorted from spleen of WT mice and were treated with LPS (100 ng/ml) for 24 hr with or without KC7F2 (HIF-1α inhibitor). Expression of HIF-1α was analyzed by Western blot analysis (A). (B) Inflammatory cytokines (IL-6 and IL-12p70) in supernatants were detected by ELISA. Data are mean ± SEM (n=5-6). **p*<0.05, ***p*<0.01, ****p*<0.001, one-tailed Student’s t-test. Data were from one representative of three independent experiments.


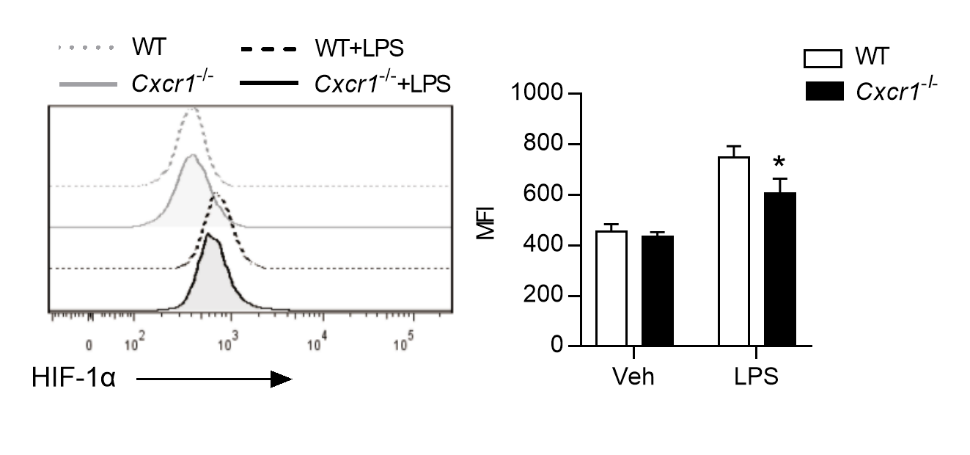


**Figure S12. CXCR1 positively regulated HIF-1α expression in DCs.** DCs were sorted from spleen of WT or *Cxcr1*^-/-^ mice and were treated with LPS (100 ng/ml) for specified time. After intracellular straining, expression of HIF-1α was analyzed by flow cytometry (left), Pooled data are presented in the right panel. Data are mean ± SEM (n=5-6). **p*<0.05, WT+LPS vs *Cxcr1*^-/-^ +LPS. one-tailed Student’s t-test. Data were from one representative of three independent experiments.


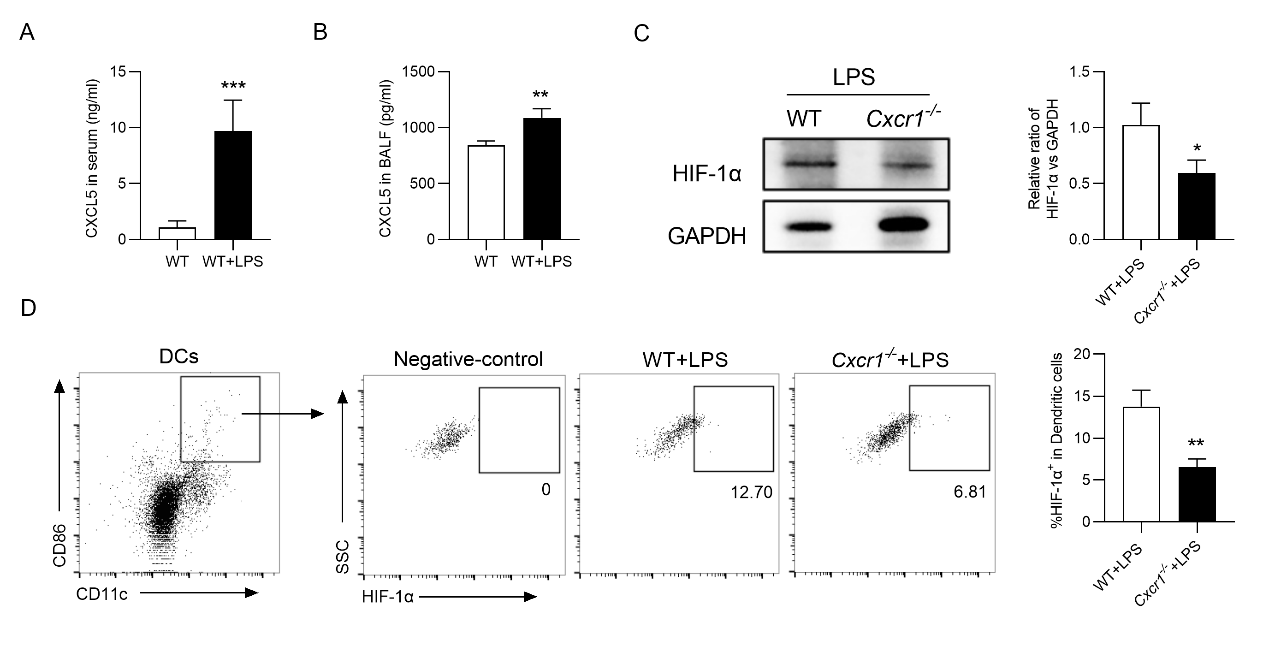
**Figure S13. CXCL5/CXCR1 axis positively regulated HIF-1α expression.** The expression of CXCL5 in serum (A) and in BALF (B) from WT mice after LPS induction for 24 hr were detected by ELISA. WT mice and *Cxcr1^-/-^* mice were stimulated by LPS for 24 hr. The protein level of HIF-1α in lung tissue was detected by western blot (C) and flow cytometry (D). (mean ± SEM; n=5-7). ^*^*p*<0.05, ***p*<0.005, ****p*<0.001. one-tailed Student’s *t*-test. Data were from one representative of three independent experiments.
