# Supplementary material for: CXCR1 drives the pathogenesis of EAE and ARDS via boosting dendritic cells-dependent inflammation
Source: Cell Death Dis. 2023 Sep 14;14(9):608. doi: 10.1038/s41419-023-06126-y (PMC10502121; doi:10.1038/s41419-023-06126-y)

Figure 5C

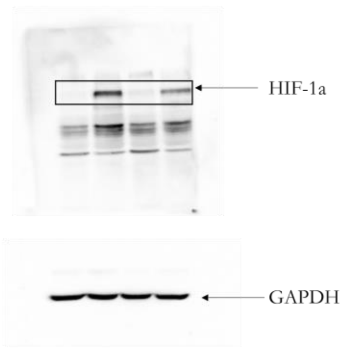

Figure 5D

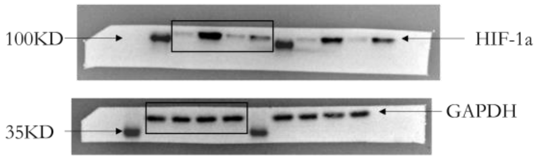

Figure 5E

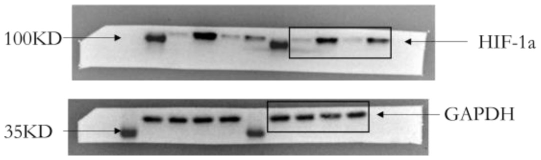

Supplement Figure 1B

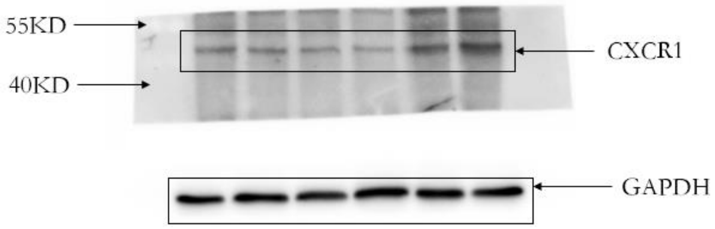

Supplement Figure 2C

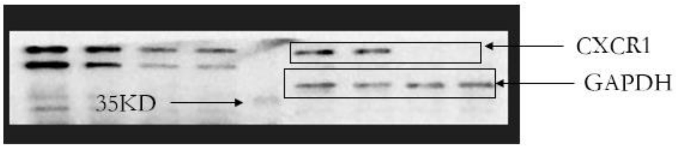

Supplement Figure 10A

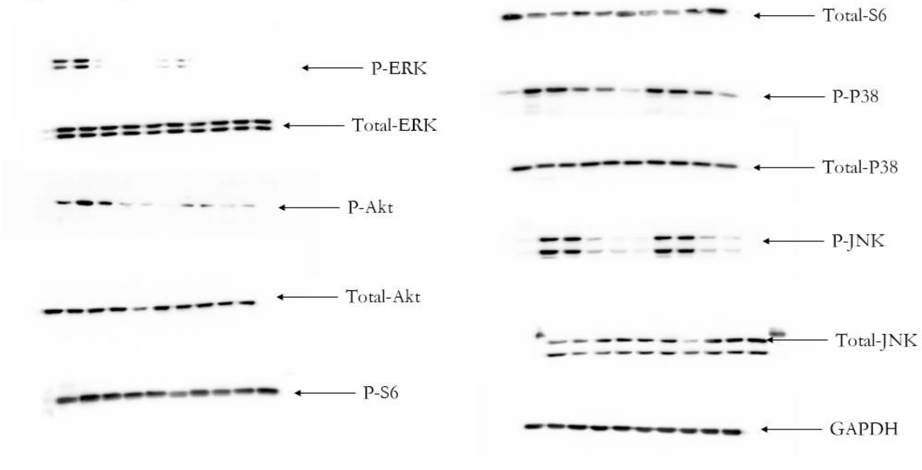

Supplement Figure 11A

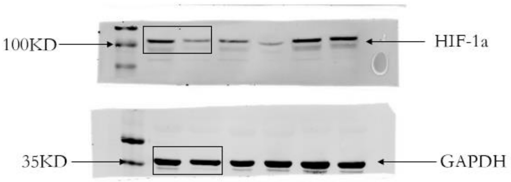

Supplement Figure 13C

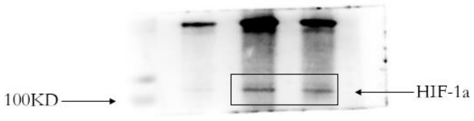

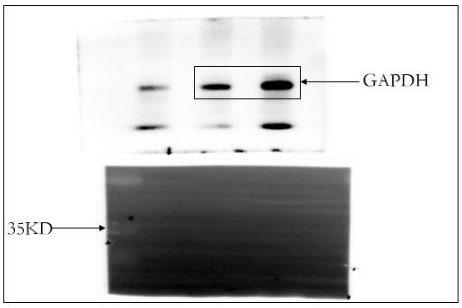

Supplement: Supplementary file 2 — Original Data File [file 41419_2023_6126_MOESM2_ESM.pdf]
